# Continuous cultivation of the lithoautotrophic nitrate‐reducing Fe(II)‐oxidizing culture KS in a chemostat bioreactor

**DOI:** 10.1111/1758-2229.13149

**Published:** 2023-03-29

**Authors:** Timm Bayer, Elizabeth J. Tomaszewski, Casey Bryce, Andreas Kappler, James M. Byrne

**Affiliations:** ^1^ Geomicrobiology Group, Center for Applied Geoscience University of Tuebingen Tuebingen Germany; ^2^ School of Earth Sciences University of Bristol Bristol UK; ^3^ Cluster of Excellence: EXC 2124: Controlling Microbes to Fight Infection Tuebingen Germany; ^4^ Present address: U.S. Geological Survey Boulder Colorado USA

## Abstract

Laboratory‐based studies on microbial Fe(II) oxidation are commonly performed for 5–10 days in small volumes with high substrate concentrations, resulting in geochemical gradients and volumetric effects caused by sampling. We used a chemostat to enable uninterrupted supply of medium and investigated autotrophic nitrate‐reducing Fe(II)‐oxidizing culture KS for 24 days. We analysed Fe‐ and N‐speciation, cell‐mineral associations, and the identity of minerals. Results were compared to batch systems (50 and 700 mL—static/shaken). The Fe(II) oxidation rate was highest in the chemostat with 7.57 mM Fe(II) d^−1^, while the extent of oxidation was similar to the other experimental setups (average oxidation of 92% of all Fe(II)). Short‐range ordered Fe(III) phases, presumably ferrihydrite, precipitated and later goethite was detected in the chemostat. The 1 mM solid phase Fe(II) remained in the chemostat, up to 15 μM of reactive nitrite was measured, and 42% of visualized cells were partially or completely mineral‐encrusted, likely caused by abiotic oxidation of Fe(II) by nitrite. Despite (partial) encrustation, cells were still viable. Our results show that even with similar oxidation rates as in batch cultures, cultivating Fe(II)‐oxidizing microorganisms under continuous conditions reveals the importance of reactive nitrogen intermediates on Fe(II) oxidation, mineral formation and cell–mineral interactions.

## INTRODUCTION

Iron (Fe) is one of the most abundant elements in the earth's crust and essential to almost all known organisms (Boyd & Ellwood, 2010; Kendall et al., 2012). Fe is commonly present as Fe(II) or Fe(III) (Cornell & Schwertmann, 2003; Kappler, Becker, & Enright, 2021) and redox cycling takes place abiotically (e.g. via light) or via microbial metabolisms (Hedrich et al., 2011; Kappler, Bryce, et al., 2021). Fe(II)‐oxidizing bacteria, at circumneutral pH, use Fe(II) as an electron donor with O_2_, CO_2_, or nitrate as electron acceptors (Bryce et al., 2018). Nitrate‐reducing Fe(II)‐oxidizing (NRFeOx) bacteria couple Fe(II) oxidation to the reduction of nitrate (NO_3_^−^) in anoxic environments (Roden, 2012; Straub et al., 1996; Weber et al., 2006). Over recent decades, NRFeOx microorganisms have been intensely studied for metabolic flexibility, microbial community composition and interactions (Bryce et al., 2018; He et al., 2016; Huang, Straub, Kappler, et al., 2021; Jakus, Blackwell, et al., 2021; Straub et al., 1996), and for their environmental impact in areas affected N‐fertilizer use (Kim et al., 2015; Visser et al., 2021; Ward et al., 2018). NRFeOx microorganisms have been found in different environments, including freshwater ponds and lakes, brackish‐waters, marine sediments, and aquifers (Emmerich et al., 2012; Finneran et al., 2002; Hafenbradl et al., 1996; Jakus, Mellage, et al., 2021; Liu, Chen, Luo, et al., 2019; Melton et al., 2012; Straub et al., 1998). NRFeOx microorganisms reduce NO_3_^−^ to nitrogen (N_2_) or ammonium (NH_4_^+^) stepwise via intermediates including NO_2_^−^, NO, and N_2_O (NO_3_^−^ → NO_2_^−^ → NO → N_2_O → N_2_) (Canfield et al., 2010; Coby et al., 2011; Straub et al., 1996; Tiedje, 1988), whereas dissimilatory nitrate reduction to ammonium (DNRA) does not involve all intermediates (NO_3_^−^ → NO_2_^−^ → NH_4_^+^). The first enriched microbial consortium capable of chemolithoautotrophic NRFeOx was described by Straub et al. (1996) as the co‐culture ‘culture KS’, with only two additional autotrophic co‐cultures since enriched (Huang, Straub, Kappler, et al., 2021; Jakus, Blackwell, et al., 2021). The term ‘co‐culture’ describes a consortium of different microorganisms, meaning it is not a pure culture. Autotrophically grown culture KS is dominated by *Gallionellaceae* sp. (96%), which is considered the main Fe(II)‐oxidizer, but also contains *Rhodanobacter* (1%) and *Bradyrhizobium* (1%) (Blöthe & Roden, 2009; He et al., 2016). Most other NRFeOx microorganisms can only be cultivated in the presence of an additional organic substrate (Benz et al., 1998; Kappler et al., 2005; Laufer et al., 2016; Liu, Chen, Li, & Li, 2019), as demonstrated, for example, for *Acidovorax* sp. BoFeN1 (Muehe et al., 2009). Despite recent studies (Dopffel et al., 2021), there is no conclusive evidence that these mixotrophic microorganisms gain energy by Fe(II) oxidation, or if they are chemodenitrifiers (Bryce et al., 2018). This suggests that Fe(II) oxidation could also be caused abiotically by reactive nitrogen species (RNS) NO_2_^−^ and NO (Kampschreur et al., 2011), which have been shown to oxidize Fe(II) (Betlach & Tiedje, 1981; Klueglein et al., 2014; Klueglein & Kappler, 2013). Abiotic oxidation of Fe(II) by RNS is described as chemodenitrification (Dhakal et al., 2013). Whenever RNS are present, NRFeOx microorganisms have to compete for Fe(II) (Klueglein et al., 2014). This makes differentiating abiotic from microbial activity challenging.

To identify, quantify, and disentangle different Fe(II) oxidation mechanisms, conditions must be as steady and controllable as possible. However, common experiments studying NRFeOx are performed in stationary batch systems with high substrate concentrations (in the order of 10s of mM) to allow cell growth and regular sampling without significant decrease of volume. High concentrations of substrates (i.e Fe(II)) can lead to toxic effects (Swanner et al., 2015) and usually result in rapid concentration changes during cultivation. Furthermore, time‐scales of batch experiments for Fe(II)‐oxidizing bacteria rarely exceed 1–2 weeks. Therefore, these experiments are limited concerning removable volume, long‐term investigation and environmental relevance. Conversely, a chemostat enables cultivation over prolonged time scales (Weusthuis et al., 1994) and provides a constant supply and steady concentrations of substrates and nutrients. Continuous addition of medium and removal of metabolites should therefore allow establishment of a steady state. Additionally, several parameters can be monitored and controlled non‐invasively (pH, dissolved oxygen, and temperature). A chemostat therefore eliminates the analytical and temporal limitations and can be used to better understand Fe(II) oxidation mechanisms like mineral transformation, fate of contaminants (Borch et al., 2009) and the coupling of biogeochemical cycles (Peiffer et al., 2021). Previous studies on Fe(II) oxidation in chemostats focused on acidophiles, as low pH environments prevent precipitation of Fe(III) minerals (Gahan et al., 2010; Ojumu & Petersen, 2011).

In this study, we established a chemostat bioreactor as cultivation method and examined growth of autotrophic NRFeOx culture KS. We compared culture KS grown in the chemostat (700 mL) for 24 days to four different batch conditions: Shaken and static in small and large volume (25 and 700 mL, respectively). We measured changes in concentrations of Fe and N species over time and analysed Fe‐minerals using μ‐x‐ray diffraction (μ‐XRD), Mössbauer spectroscopy, and x‐ray absorption spectroscopy (XAS), and visualized cell–mineral‐associations with scanning electron microscopy (SEM).

## EXPERIMENTAL PROCEDURES

### Bacterial strain, pre‐cultivation, and growth conditions

Culture KS, obtained from the culture collection of the Tuebingen Geomicrobiology Group, was first grown for 7 days and then transferred to fresh medium and grown for another 7 days, both times under autotrophic conditions with 10 mM Fe(II) and 4 mM NO_3_^−^ on bicarbonate‐buffered (22 mM) basal medium, as previously described (Tominski et al., 2018). All experiments were performed with 10 mM Fe(II) as FeCl_2_ and 4 mM NO_3_^−^ as NaNO_3_ for batch system. For the chemostat, the same medium was continuously supplied (see below). All experiments were performed at 28°C and pH 7.0. In the chemostat bioreactor, the conditions were stable (pH ± 0.25 and T ± 0.5°C, logged data Figure S2).

### Setup of the chemostat and batch experiments

**FIGURE 1:**
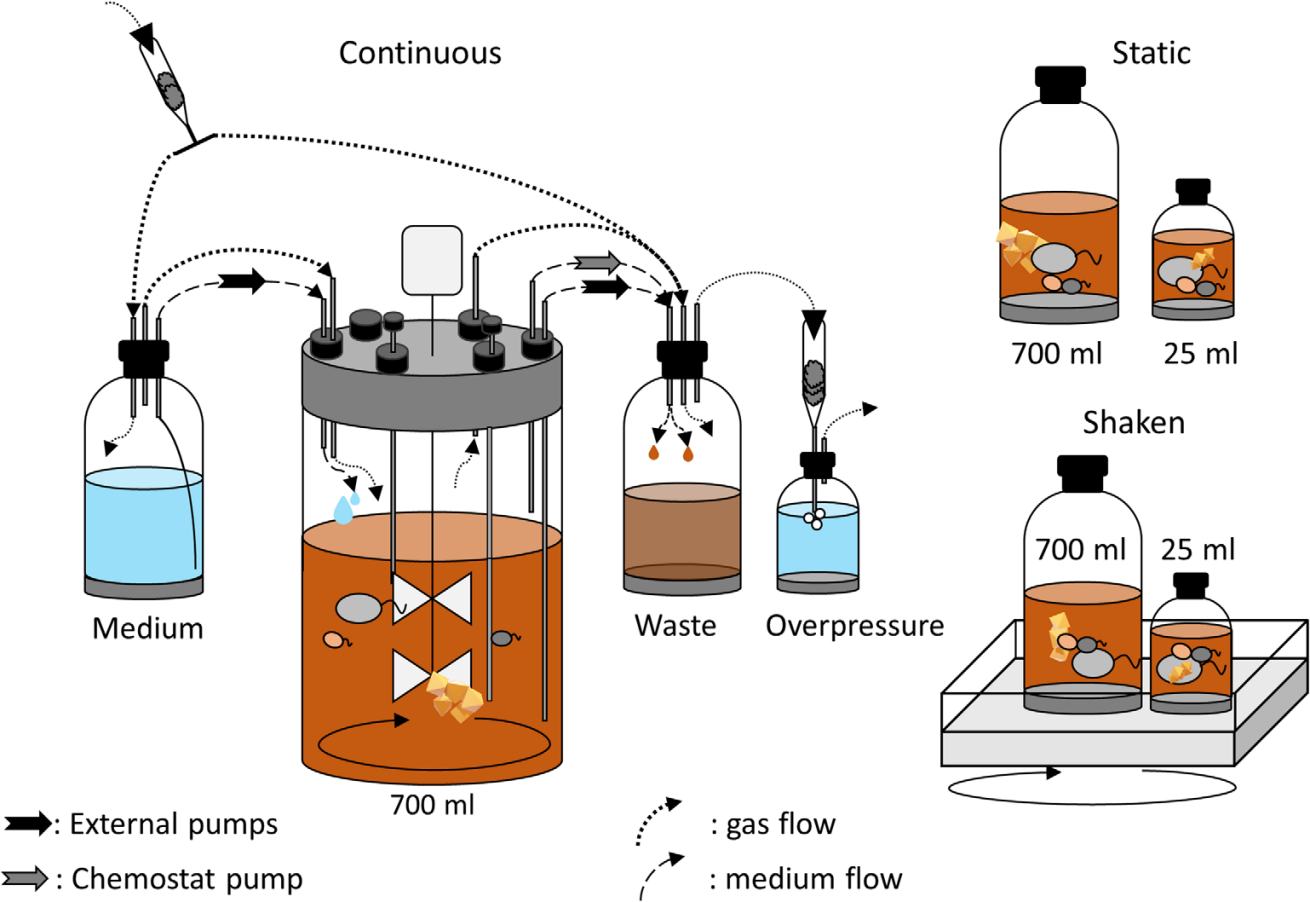
Schematic overview of the setup of the chemostat. Arrows indicate flow of gas or liquids. All parts were connected and inoculated under sterile conditions. The chemostat chamber and all connected bottles were flushed with N_2_/CO_2_ to keep the system anoxic and to provide inorganic carbon. Control experiments were conducted in small and big volumes (25 and 700 mL medium, respectively) in static or shaken (50 rpm) conditions.

In the chemostat, culture KS was cultivated in 700 mL inside a glass vessel (New Brunswick Scientific, USA) covered with a dense black cloth to prevent photo‐oxidation of Fe(II) (Figures 1 and S1). The growth medium, reaction chamber and waste‐collection systems were interconnected and continuously flushed with N_2_/CO_2_ (90:10 v/v %) at 10 mbar overpressure, to maintain anoxic conditions and provide inorganic carbon (Figure 1, Figure S1). Oxygen was measured with a sensor (Mettler InPro 6800 Series, Mettler‐Toledo AG, Urdorf, Switzerland, detection limit 6 ppb) and an oxygen sensitive foil with a Fibox 3 optode oxygen measurement device (Presens, Germany). After medium addition, the glass vessel was visually inspected for 48 h for precipitates in a brownish colour that would indicate abiotic Fe(II) oxidation by O_2_. The expected presence of only greyish precipitates due to medium composition (Fe(II)‐minerals) during this abiotic pre‐incubation confirmed the absence of oxygen. Then cells were added and to ensure that enough viable cells were present despite low inoculum, the pumping was started 24 h after inoculation with a low dilution rate of 15 mL h^−1^. Temperature, pH, and DO were measured in situ (Figures S1 and S2). For pumping in and out of the reaction vessel, external peristaltic pumps (MS‐MC/CA 4, Ismatec, Germany) were used. The output rate was set slightly lower than the input. A conductivity sensor was utilized to keep the volume between 700 and 710 mL. Further information on setting up the chemostat can be found in the supplementary information (SI). Due to the complexity of setting up the chemostat, a single run was performed for this study. An additional run showing reproducibility is presented in the SI. Batch experiments were conducted in biological triplicates in liquid volumes of either 25 mL or 700 mL either static or shaken at 50 rpm (Figure 1). An abiotic control was performed for all batch experiments. All biotic experiments were inoculated with 1% (v/v) of a pre‐grown culture KS (as described).

### Geochemical analyses and Fe(II) oxidation rates

Samples were centrifuged in a glovebox (100% N_2_, MBraun Germany) and split for measurements of the pellet and supernatant. Fe(II) and total Fe were determined using the spectrophotometric ferrozine assay (Stookey, 1970). NO_3_^−^ and NO_2_^−^ were measured using a continuous‐flow analyser (Seal Analytical; Norderstedt, Germany). The maximum Fe(II) oxidation rates in batch setups were determined from the difference between Fe(II) concentrations across consecutive timepoints. The calculated Fe(II) oxidation rate in the chemostat was adjusted to account for the continuous addition of Fe(II) (Figure S3). Detailed descriptions are provided in the SI.

### Mineralogical and microscopic analyses

For Mössbauer spectroscopy, minerals were collected by filtration through a 0.45 μm pore‐size syringe filter (Millipore membrane) in a glovebox, embedded between two layers of Kapton tape foil and stored frozen (−20°C) and anoxically until analysis. Collected sample spectra were analysed with respect to the isomer shift (δ) values and the quadrupole splitting (ΔE_Q_) and the Gaussian width (standard deviation) of the ΔE_Q_ was used to account for line broadening until the fit was reasonable. For μ‐XRD, samples were collected and air dried in an Eppendorf tube in an oven at 27°C inside an anoxic glovebox. For x‐ray adsorption spectroscopy (XAS), an anoxically dried sample taken after 40 days (no geochemistry measured) was diluted with polyvinylpyrrolidone and pressed into 7‐mm pellets using a KBr pellet press (International Crystal). The pellet was anoxically sealed in Kapton tape. X‐ray absorption spectroscopy was performed at the Advanced Photon Source (APS) Materials Research Collaborative Access Team (MRCAT) beamline 10‐ID‐B at Argonne National Laboratory (Segre et al., 2000). SIXpack (Webb, 2005) software was used to perform linear combination fitting (LCF) analysis after spectral processing (Supplementary Information S1). Samples for scanning electron microscopy were fixed in 2.5% glutaraldehyde overnight at 4°C. Micrographs were collected using a JEOL JSM‐6500F field emission SEM with a Schottky‐field‐emitter at a working distance of approximately 10 mm at the Centre for Light‐Matter Interaction, Sensor & Analytics (LISA^+^), University of Tuebingen. Detailed descriptions available in the SI.

## RESULTS AND DISCUSSION

### Fe(II) oxidation and NO_2_ ^−^ production during growth of culture KS

**FIGURE 2:**
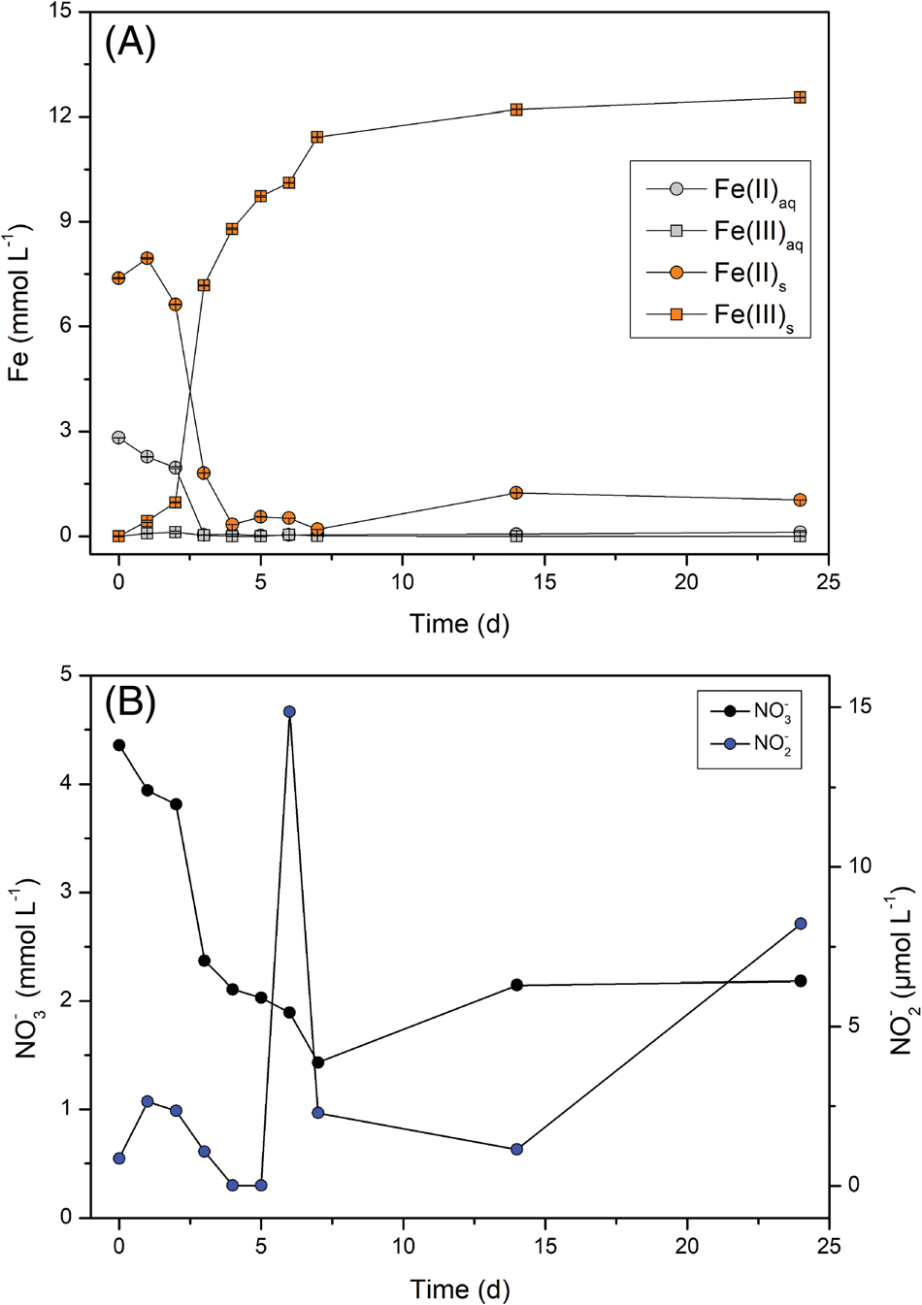
Changes of Fe(II)/Fe(III) (A) and NO_3_^−^/NO_2_^−^ (B) concentrations in the chemostat during autotrophic cultivation of culture KS for 25 days. Fe: Circles show Fe(II) species while squares show Fe(III) species for aqueous (aq—grey) and solid phase (s—orange) iron (mmol L^−1^). Error bars (not visible if smaller than symbol) represent measurement error (standard deviation) from ferrozine replicate measurements. NO_3_^−^: black circles indicate NO_3_^−^ concentrations (mmol L^−1^—left y axis) while blue circles show NO_2_^−^ concentrations (μmol L^−1^—right y axis).

We quantified concentrations of Fe(II), Fe(T), as well as NO_3_^−^ and NO_2_^−^ during autotrophic growth of culture KS (Figure 2, Figures S4–S11). Results indicated that Fe was initially only present as Fe(II), with the majority (68%) as solid phase, as expected due to precipitation of Fe(II) minerals (siderite and vivianite) during medium preparation (Hegler et al., 2008; Miot et al., 2009; Nordhoff et al., 2017; Tominski et al., 2018). Most importantly for the chemostat, as the medium was pumped in 2 days before inoculation, the absence of Fe(III) thereafter confirmed anoxia. After a2‐day lag phase after inoculation, rapid Fe(II) oxidation occurred. In the chemostat, no Fe(II)_aq_ could be measured after Day 3, even though fresh medium containing 2.8 mM Fe(II)_aq_ was continuously pumped in, suggesting high microbial activity. At Day 3, a low concentration of Fe(II)_s_ (1.88 mM) was still measured, while at Day 4, the Fe(II)_s_ concentration decreased to 0.33 mM. This delay between Fe(II)_aq_ and Fe(II)_s_ consumption implies that easily accessible Fe(II)_aq_ was preferably oxidized. The absence of Fe(II)_aq_ measured beyond Day 4, despite continued addition, along with microscopy and nitrogen speciation data (discussed below), suggest microbial activity until the end of the experiment. The aqueous chemical analyses of the chemostat were comparable to the results collected from the batch experiments: Fe(II)_aq_ was quickly consumed prior to Fe(II)_s_, that remained despite available NO_3_^−^ (Fe(II)_s_ at the end of experiments: 50 mL, static: 1 mM, 50 mL shaken: 0.3 mM, 700 mL static: 0.7 mM, 700 mL shaken: 0.56 mM, chemostat: 1 mM). These results agree with previous studies (Blöthe & Roden, 2009; Nordhoff et al., 2017; Tominski et al., 2018). We therefore hypothesize that culture KS is not capable of fully oxidizing all solid‐phase Fe(II). We were unable to identify this remaining Fe(II) mineral phase since neither μ‐XRD nor Mössbauer measurements were conclusive. However, Tominski et al. (2018) described that culture KS was not capable of oxidizing vivianite, and thus, we suggest a Fe(II)‐phosphate mineral could be the remaining Fe(II)_s_. For the chemostat, Fe geochemistry data first suggested that steady state was reached at Day 7, as we could only detect little Fe(II)_s_ (0.2 mM) that seemed to be constant after Day 4, and additionally no Fe(II)_aq_ was measured. At Day 14, however, more Fe(II)_s_ was measured (1.24 mM), which suggests that the steady state was only achieved between days 7 and 14 in the chemostat (Figure 2). We suggest that at this time culture KS fully adapted to the conditions and quickly oxidized all bioavailable Fe(II) that was pumped into the chemostat. It is likely that the oxidation rates could have been even greater in the chemostat bioreactor if more Fe(II) was provided. In all systems, the Fe(II) oxidation occurred simultaneously with NO_3_^−^ reduction, decreasing from approximately 4 mM to around 2 mM, and finally stabilizing at 2.2 mM for the chemostat. NO_3_^−^ reduction approached the expected extent based on the stoichiometric ratio of Fe to NO_3_^−^. Fe(II) oxidation yields one electron, reduction of NO_3_^−^ to N_2_ requires five electrons. Therefore, 10 mM Fe(II) (sum of Fe(II)_aq_ and Fe(II)_s_) could be oxidized by roughly 2 mM NO_3_^−^, as shown by our data (Figure 2, Figures S4–S11). Averaged across all performed experiments (chemostat and all batch) 2.41 ± 0.28 mM of NO_3_^−^ was reduced. We propose that the uptake of electrons by Fe(II)‐oxidizing bacteria can lead to intracellularly stored electrons and a reduced redox environment (Guzman et al., 2019) and therefore explain the surplus of reduced NO_3_^−^, even though some of the electrons must be used for CO_2_ fixation. We propose that electrons from dead biomass could additionally serve as a source of electrons. Nitrate reduction with these electrons could explain the deviation from the expected ratio towards more NO_3_^−^ reduction. Interestingly, we detected approximately 15 μM of the reactive nitrogen species nitrite (NO_2_^−^) in the aqueous phase of the chemostat bioreactor. Formation of nitrite has, to the best of our knowledge, not been previously reported for culture KS. The detection of NO_2_^−^ occurred after the fastest rate of NO_3_^−^ reduction (between Days 3 and 6). NO_2_^−^ is very reactive and will rapidly transform to NO and NO_2_, and react abiotically with Fe(II) to form N_2_O during chemodenitrification (Dhakal et al., 2013; Klueglein & Kappler, 2013). We observed a slight delay between highest NO_3_^−^ consumption and NO_2_^−^ formation of approximately 3 days. We propose that this shift is caused by NO_2_^−^ consumption (biotically) and reactivity (chemodenitrification). Batch experiments showed a similar behaviour in terms of NO_3_^−^ reduction (Figures S8–S11). We detected NO_2_^−^ in the batch experiments, showing that it was not only produced in the continuous system. The nitrite concentration was dependent on the experimental setup and increased in the following order (mean values ± standard deviation): 50 mL static (12.5 ± 3.1 μM), 50 mL shaking (18.7 ± 6.2 μM), 700 mL shaking (37.5 ± 12.3 μM), 700 mL static (234 ± 102.4 μM) (Figures S8–S11). The highest concentration of NO_2_^−^ (234 μM) was detected in the 700‐mL static bottles. We speculate that NO_2_^−^ was consumed more rapidly in the well‐mixed systems and the systems of small volume, since there was less diffusion limitation (agitation) of substrates (small volume). In the mixed setups, all compounds were homogeneously distributed and hence geochemical gradients not expected, which could have hindered microbial activity and abiotic reactivity between Fe(II) and NO_2_^−^, causing accumulation of the latter. To unravel this, expression and activity levels of nitrate and nitrite reductase could be studied in the chemostat.

### Fe(II) oxidation rates in different cultivation conditions

The Fe(II)_s_ oxidation rate was greater than for Fe(II)_aq_ in all setups. For the chemostat Fe(II)_aq_ oxidation rate was 2.36 mM d^−1^ compared to 5.21 mM d^−1^ for Fe(II)_s_ (see SI). The total oxidation rate (Fe(II)_s+aq_) in the chemostat was the highest for all experiments. All rates are listed in Table 1. We applied an unpaired *t*‐test to determine whether there was any statistical significance for which multiple replicated experiments (no replicates were available for the chemostat). Fe(II)_s_ and Fe(II)_aq_ oxidation was significantly different for all treatments (*p* = 0.004). Additionally, we observed a significant difference between the solid phase Fe(II) oxidation rate between different volumes (*p* = 0.03) (Table S2).

**TABLE 1:** Maximum iron oxidation rates calculated for aqueous iron(II) (Fe(II)_aq_) and solid phase iron(II) (Fe(II)_s_), for 25 and 700 mL batch experiments (static and shaken) and the chemostat.

Setup	Fe(II) oxidation (mM d^−1^)	
	Aqueous Fe(II)	Solid phase Fe(II)
25 mL static	1.95 ± 0.14	3.28 ± 1.38
25 mL shaken	1.82 ± 0.39	4.35 ± 0.66
700 mL static	2.57 ± 0.41	2.68 ± 1.11
700 mL shaken	2.03 ± 0.51	2.99 ± 0.59
Chemostat	2.36	5.21

*Note*: Errors correspond to 1 standard deviation (1σ) from the mean of biological replicates of the batch experiments. Biological replicates were not available for the chemostat, so no error is reported.

### Fe mineral formation and transformation

**FIGURE 3:**
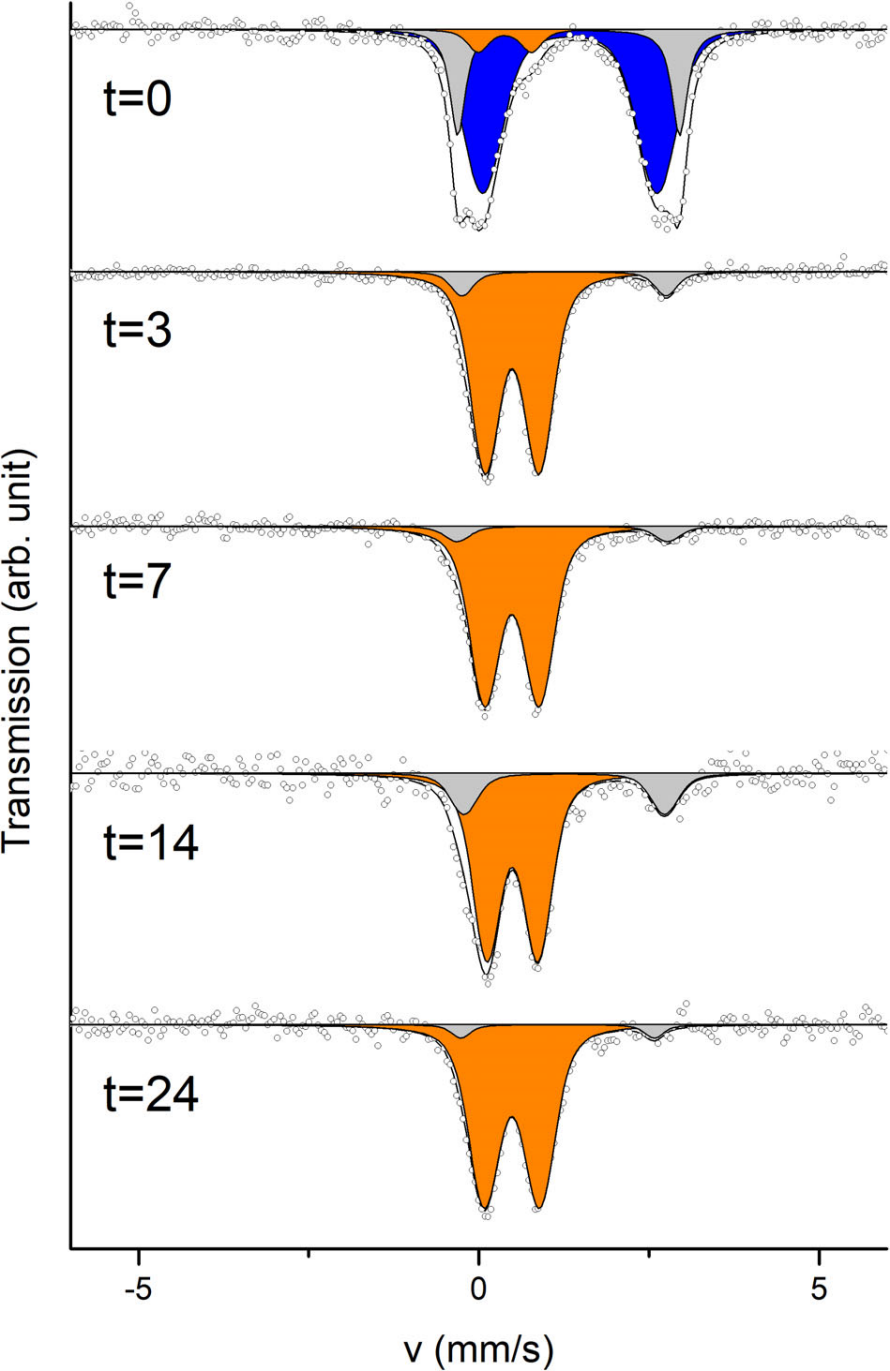
^57^Fe Mössbauer spectra of anoxically filtered mineral precipitates formed in the chemostat during autotrophic NRFeOx by culture KS (collected after 0, 3, 7, 14, and 24 days of continuous cultivation in the chemostat).

^57^Fe Mössbauer spectroscopy revealed that the starting sample was dominated by Fe(II), though the fit was incomplete without an Fe(III) doublet which accounted for 6.0% of the spectral area (Figure 3, Table S1). This Fe(III) originated from the inoculum. The initial Fe(II) component most likely consisted of a combination of siderite (FeCO_3_) and vivianite (Fe_3_(PO_4_)_2_·8(H_2_O)), which were expected to precipitate immediately after addition of dissolved FeCl_2_ medium, as discussed before. The precipitates in the chemostat were dominated by Fe(III) at Day 3 (90.8% relative abundance) and the remaining 9.2% was Fe(II). A Fe(II) component of up to 18.3% relative abundance was still measured at Day 14. This increase of spectral area of Fe(II) agrees with the increased concentration of Fe(II) measured with ferrozine. The final sample taken after 24 days was dominated by a superparamagnetic Fe(III) doublet with 94.9% spectral area. The remaining 5.1% corresponded to a Fe(II) doublet. The detected Fe(III) component is most likely a short‐range ordered Fe(III) (oxyhydr)oxide such as ferrihydrite, though without measuring at lower temperature confirming it is not possible. Previous experiments with culture KS have revealed similar products, that is (short‐range ordered) ferrihydrite (Nordhoff et al., 2017). This suggests, unexpectedly, that the stirring and continuity of the chemostat did not lead to major differences in mineral precipitation despite having the highest total oxidation rate. The Fe(II) phase could resemble vivianite, as suggested by previous reports, but this could not be confirmed without further measurements. Oxidized Fe(III) minerals continuously accumulated in the chemostat bioreactor up to concentrations of 12.5 mM, higher than the added concentration (Figure 2). This increase was caused by Fe(III) (oxyhydr)oxide settling to the bottom of the reactor, while the outflow tube was placed well above the mineral layer. We anticipated that given longer incubation time, minerals of higher crystallinity such as goethite or lepidocrocite may form (Han et al., 2020; Hansel et al., 2003). μ‐XRD patterns (Figure S12) of samples indicated the presence of crystalline mineral reflections (2θ of 36° and 53°) in all samples which most likely correspond to dried salts from the medium and reflections from the sample holder (2θ of 52° and 65°). The only Fe mineral detected in any of the samples was found in a sample collected from the chemostat at Day 14, with reflections most closely matching vivianite (Figure S12C), as expected. The absence of any other reflections corresponding to Fe minerals suggests short‐range ordered Fe mineral such as ferrihydrite, which does not typically yield a clear diffraction pattern with the x‐ray source used here (see samples 25 mL shaken [Figure S12A], and 700 mL shaken [Figure S12B]). The diffractogram of sample chem2 (Figure S13D, sample from chemostat at timepoint 24) exhibited reflections which most closely matched wuestite. However, the presence of this mineral is unlikely because wuestite is typically found in more reducing conditions (Cornell & Schwertmann, 2003) or at higher temperatures (Jette & Foote, 1933). This additional reflection of sample taken form the chemostat at 24 days (Figure S12D) shows that more crystalline phases formed over time, further confirmed by XAS measurements. After the final sampling of the experiment, the chemostat was still maintained continuously for another 20 days. At Day 40, a sample of the very bottom of the bioreactor vessel was taken and prepared for XAS measurements. The results (Figures S13 and S14) showed crystalline Fe(III) phases: 48.7 mol% goethite and 11.6 mol% lepidocrocite. Still, around 40 mol% of detected Fe(III) phases were determined to be ferrihydrite in this sample (Table S14). Previous studies have shown that low concentrations of Fe(II) can cause transformation to lepidocrocite and goethite (Hansel et al., 2005). Since we continuously added Fe(II) to the chemostat and also measured some leftover Fe(II)_s_ we suggest transformation to a greater extent in the chemostat than in the batch systems.

### Cell encrustation during autotrophic NRFeOx visualized by SEM

**FIGURE 4:**
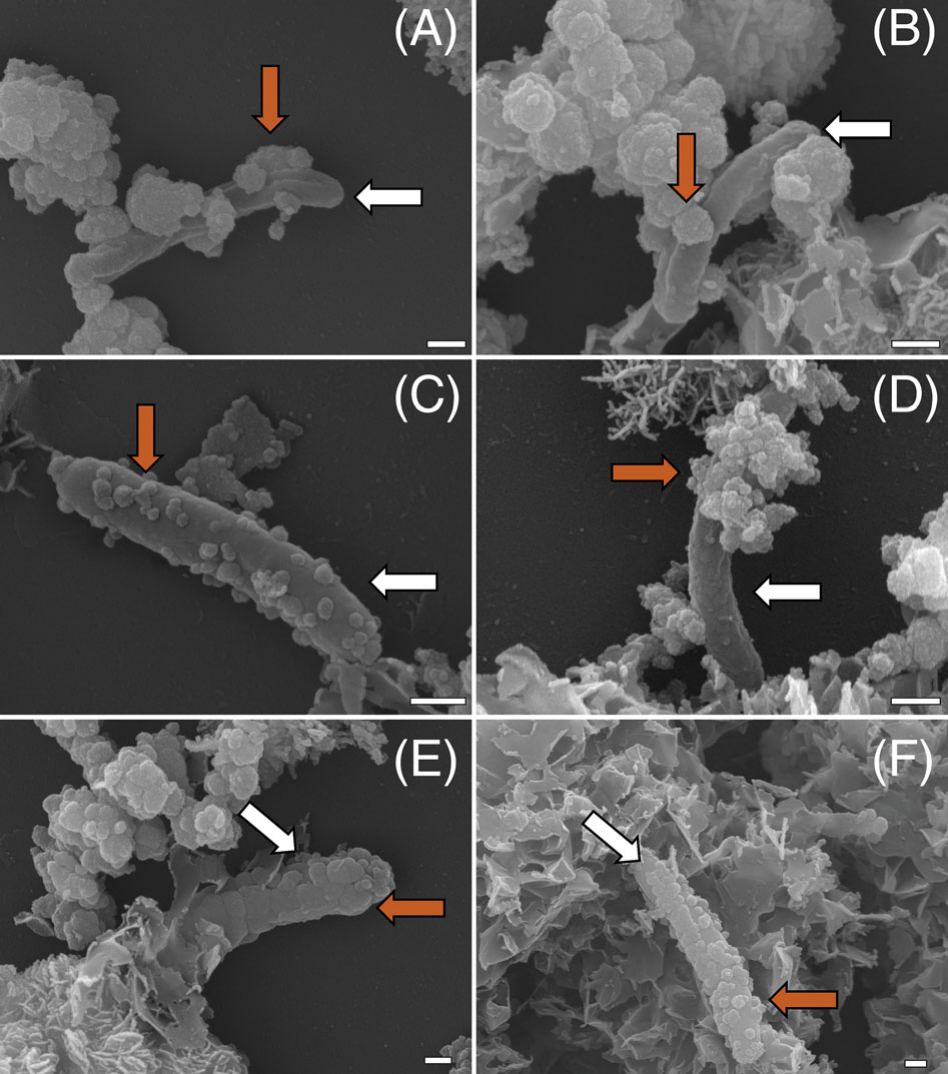
Scanning electron micrographs of culture KS grown autotrophically in the chemostat under continuous cultivation. Micrographs were collected at 15 kV acceleration voltage. (A–C) Cells that are associated with a small extent with minerals. (D–F) Cells that become encrusted with iron minerals. White scale bar represents 200 nm. White arrows indicate not encrusted cell surface and orange arrows point to different degrees of encrustation.

The interaction between Fe minerals and cells from culture KS was visualized using SEM (Figure 4), focusing mainly on the chemostat, after confirming that cells showed the same patterns for all experiments. The micrographs revealed that many cells were associated with Fe minerals to varying degrees (Figure S15). Of all imaged cells (*n* = 78), 58% were free of iron minerals, 31% were closely associated/partly encrusted with iron minerals, and 11% were completely encrusted with Fe minerals. Based on the morphology of the cells, it is likely that these cells are *Gallionellaceae* sp. (Nordhoff et al., 2017). This is also supported by previous work, where *Gallionellaceae* sp. was reported as dominating in autotrophic conditions (Tominski et al., 2018). Until now, the absence of encrustation by culture KS, alongside with the lack of detection of NO_2_^−^, was used to support the hypothesis of exclusively enzymatic Fe(II) oxidation (Nordhoff et al., 2017; Straub et al., 1996; Tominski et al., 2018). In contrast to this, we show that 42% of all imaged cells were at least partly associated with minerals and that 11% were completely encrusted and therefore suggest that not all oxidation is enzymatic. Since we quantified NO_2_^−^ (Figures 2 and S8–S10) and saw 42% of all counted cells to be at least partly encrusted, abiotic oxidation of Fe(II) (chemodenitrification) should be considered during autotrophic NRFeOx by culture KS. These findings suggest that future research needs to account for these processes when studying NRFeOx, especially when calculating turnover rates of Fe(II) and NO_3_^−^/NO_2_^−^. In cultures of the NRFeOx *Acidovorax sp*. BoFeN1, encrustation by Fe(III) mineral precipitates was shown to occur as a result of abiotic Fe(II) oxidation by NO_2_^−^ (Klueglein et al., 2014; Klueglein & Kappler, 2013; Schmid et al., 2014). We propose that this abiotic oxidation due to NO_2_^−^ or NO also occurred in in this study. The main Fe(II)‐oxidizer of culture KS, *Gallionellaceae* sp., is suggested to be unable to perform NO‐reduction enzymatically and hence relies on other members of the enrichment culture for NO‐detoxification (He et al., 2016; Huang, Straub, Blackwell, et al., 2021). Prolonged presence of NO, and possibly NO_2_^−^, could have caused the encrustation, possibly limiting the access to substrates and cell growth and division. We speculate that the extent of encrustation varies depending on the age of individual cells, the amount of RNS produced, and the abundance of flanking community members, which are essential for RNS removal. Additionally, intact surface areas of dead cells could serve as a template for mineral‐precipitation similar to the way that twisted stalk forming Fe(II)‐oxidizing bacteria provide a template for mineral precipitation (Chan et al., 2011). This dead‐cell‐encrustation would, however, limit organic compounds from dead cells that would otherwise be available from lysed cells. If this process is happening in anoxic, NO_3_^−^‐rich aquatic systems, where Fe(II) is oxidized, cell encrustation would effectively trap organic carbon (cells and content) within this system. In the chemostat, even after 24 days of continuous cultivation, geochemical measurements, fluorescence microscopy (Figure S16) and SEM micrographs (Figure 4) suggested viable cells. We therefore propose that the chemostat bioreactors' design fulfils the requirements to study NRFeOx over extended periods of time.

### Advantages and challenges of continuous cultivation in a chemostat

To circumvent the limitations of small‐scale batch experiments, we established a chemostat for continuous cultivation of Fe(II)‐oxidizing bacteria, here for the autotrophic NRFeOx culture KS. Compared to our experimental control setups, representing common batch cultivation methods, and previously published data (Nordhoff et al., 2017; Tominski et al., 2018), the chemostat showed comparable results for Fe(II) oxidation rates and Fe mineralogy. The total Fe(II) oxidation rate was highest in the chemostat, as metabolizing cells were continuously provided Fe(II). Additionally, we showed mineral transformation in the chemostat. Ostwald ripening is a well‐described process for minerals and time‐dependent ripening of ferrihydrite to more crystalline phases like goethite and Fe(II) catalysed transformations were previously described (Burleson & Penn, 2006; Cornell & Schwertmann, 2003; Tomaszewski et al., 2017). This could have great influence for long‐term experiments, as mineral surfaces greatly influence nutrient availability (Gu et al., 1994) and heterogeneous Fe(II) oxidation (Hansen et al., 1994; Sørensen & Thorling, 1991). Maintaining O_2_‐free conditions despite connecting multiple tubes (Figure S1) was achieved by applying a slight overpressure of N_2_/CO_2_ and proper attachment and sealing of all connections. High mixing velocities were avoided to ensure cell viability while homogenizing the volume as well as possible. Chemostats are built for long‐term experiments, and hence performing several replicated runs for extended periods of time is challenging, as setting up takes significantly more time than batch experiments and is prone to difficulties. Additionally, parallel setups are only possible with multiple chemostats. Lack of replicates is an obvious disadvantage compared to the smaller batch setups, where multiple replicates can be easily performed in parallel. Results from an additional run are provided in Figure S17 and Table S3, though operational differences mean that direct comparison between these data and those reported above is not possible. Key aspects that should be considered when working anoxically and under sterile conditions with the chemostat are (1) sterilization and sterile connection of large vessels, (2) sterile addition of anoxic medium and inoculum of bacteria, and (3) maintaining anoxic conditions during long‐term cultivation and sampling. The chemostat's bioreactor allows to maintain, change, and automatically adjust key parameters like pH and temperature. Additionally, low concentrations of nutrients can be continuously provided. Since Fe precipitates can block the tubing, the system could be refined for even more homogeneousness and easier sampling. In‐depth descriptions are available in the SI. The chemostat setup presented here was optimized for the cultivation of enrichment culture KS. We suggest it could be applied for different types of Fe(II)‐oxidizing bacteria. However, predicting behaviour of bacteria in a new cultivation vessel is uncertain, and hence we suggest performing all controls as described here. Despite these challenges and the limited replication possibilities, the chemostat bioreactor is a valuable tool to investigate microbial activity under continuous conditions. Ideally, the chemostat could be applied for in‐depth studies to obtain valuable data such as growth yield and rate and additionally, steady state cells would be ideal for expression studies.

## CONCLUSION

We have successfully established a chemostat bioreactor system for investigating NRFeOx. By using it, we have for the first time measured NO_2_^−^ in autotrophic culture KS. NO_2_^−^ can abiotically oxidize Fe(II), possibly influencing rates and competing with the enzymatic oxidation. We therefore suggest consideration of chemodenitrification during autotrophic NRFeOx. For future studies, we propose to carefully follow NO_2_^−^, NO, and N_2_O formation when studying NRFeOx, for a better understanding of the production of and impact on Fe(II) oxidation. We used SEM to show associations of cells with Fe minerals, including complete cell encrustation. We propose that encrustation might be promoted by abiotic oxidation of Fe(II) due to NO_2_^−^. Geochemical data collected during growth of culture KS in the chemostat showed that we successfully established a growth vessel to study NRFeOx under continuous conditions. We showed microbial activity for at least 24 days, allowing us to better understand processes that could be happening in a continuous and anoxic environment, that these microbes could be inhabiting in nature. Iron oxidation rates calculated for the chemostat were in the same order of magnitude compared to batch studies. The chemostat however showed the highest total oxidation rate. We focused on NRFeOx culture KS, though the chemostat system can be adapted to phototrophic Fe(II)‐oxidizers by providing a light source and for microaerophilic Fe(II)‐oxidizers by bubbling a defined gas mixture into the system. Overall, the chemostat is a powerful tool to study Fe(II)‐oxidizing microorganisms in continuous cultivation with constant supply of nutrients and substrates. This enabled studying Fe(II)‐oxidizing culture KS for a prolonged time and allowed us to detect so far undescribed processes of nitrite formation and cell encrustation.

## AUTHOR CONTRIBUTIONS

**Timm Bayer:** Conceptualization (equal); data curation (equal); formal analysis (equal); investigation (equal); methodology (equal); visualization (equal); writing – original draft (equal); writing – review and editing (equal). **Elizabeth J. Tomaszewski:** Formal analysis (equal); investigation (equal); writing – original draft (equal); writing – review and editing (equal). **Casey Bryce:** Methodology (equal); supervision (supporting); writing – original draft (equal); writing – review and editing (equal). **Andreas Kappler:** Conceptualization (equal); funding acquisition (equal); supervision (supporting); writing – original draft (equal); writing – review and editing (equal). **James M. Byrne:** Conceptualization (equal); formal analysis (equal); methodology (equal); supervision (lead); visualization (equal); writing – original draft (equal); writing – review and editing (equal).

## CONFLICT OF INTEREST STATEMENT

The authors declare no known conflict of interest.

## Supporting information


**Data S1.** Supporting Information.

**Data S2.** Supporting Information.

## Data Availability

The authors confirm that the data supporting the findings of this study are available within the article and its supplementary materials.

## References

[emi413149-bib-0001] Benz, M. , Brune, A. & Schink, B. (1998) Anaerobic and aerobic oxidation of ferrous iron at neutral pH by chemoheterotrophic nitrate‐reducing bacteria. Archives of Microbiology, 169, 159–165. 9446687 10.1007/s002030050555

[emi413149-bib-0002] Betlach, M.R. & Tiedje, J.M. (1981) Kinetic explanation for accumulation of nitrite, nitric oxide, and nitrous oxide during bacterial denitrification. Applied and Environmental Microbiology, 42, 1074–1084. 16345900 10.1128/aem.42.6.1074-1084.1981 PMC244157

[emi413149-bib-0003] Blöthe, M. & Roden, E.E. (2009) Composition and activity of an autotrophic Fe(II)‐oxidizing, nitrate‐reducing enrichment culture. Applied and Environmental Microbiology, 75, 6937–6940. 19749073 10.1128/AEM.01742-09 PMC2772415

[emi413149-bib-0004] Borch, T. , Kretzschmar, R. , Kappler, A. , Cappellen, P.V. , Ginder‐Vogel, M. , Voegelin, A. et al. (2009) Biogeochemical redox processes and their impact on contaminant dynamics. Environmental Science & Technology, 44, 15–23. 10.1021/es9026248 20000681

[emi413149-bib-0005] Boyd, P. & Ellwood, M. (2010) The biogeochemical cycle of iron in the ocean. Nature Geoscience, 3, 675–682.

[emi413149-bib-0006] Bryce, C. , Blackwell, N. , Schmidt, C. , Otte, J. , Huang, Y.M. , Kleindienst, S. et al. (2018) Microbial anaerobic Fe(II) oxidation–ecology, mechanisms and environmental implications. Environmental Microbiology, 20, 3462–3483. 30058270 10.1111/1462-2920.14328

[emi413149-bib-0007] Burleson, D.J. & Penn, R.L. (2006) Two‐step growth of goethite from ferrihydrite. Langmuir, 22, 402–409. 16378452 10.1021/la051883g

[emi413149-bib-0008] Canfield, D.E. , Glazer, A.N. & Falkowski, P.G. (2010) The evolution and future of Earth's nitrogen cycle. Science, 330, 192–196. 20929768 10.1126/science.1186120

[emi413149-bib-0009] Chan, C.S. , Fakra, S.C. , Emerson, D. , Fleming, E.J. & Edwards, K.J. (2011) Lithotrophic iron‐oxidizing bacteria produce organic stalks to control mineral growth: implications for biosignature formation. The ISME Journal, 5, 717–727. 21107443 10.1038/ismej.2010.173 PMC3105749

[emi413149-bib-0010] Coby, A.J. , Picardal, F. , Shelobolina, E. , Xu, H. & Roden, E.E. (2011) Repeated anaerobic microbial redox cycling of iron. Applied and Environmental Microbiology, 77, 6036–6042. 21742920 10.1128/AEM.00276-11 PMC3165426

[emi413149-bib-0011] Cornell, R.M. & Schwertmann, U. (2003) The iron oxides: structure, properties, reactions, occurrences and uses, Vol. 664. Weinheim: Wiley‐vch.

[emi413149-bib-0013] Dhakal, P. , Matocha, C. , Huggins, F. & Vandiviere, M. (2013) Nitrite reactivity with magnetite. Environmental Science & Technology, 47, 6206–6213. 23662623 10.1021/es304011w

[emi413149-bib-0014] Dopffel, N. , Jamieson, J. , Bryce, C. , Joshi, P. , Mansor, M. , Siade, A. et al. (2021) Temperature dependence of nitrate‐reducing Fe(II) oxidation by acidovorax strain BoFeN1–evaluating the role of enzymatic vs. abiotic Fe (II) oxidation by nitrite. FEMS Microbiology Ecology, 97, fiab155. 10.1093/femsec/fiab155 34849752

[emi413149-bib-0018] Emmerich, M. , Bhansali, A. , Lösekann‐Behrens, T. , Schröder, C. , Kappler, A. & Behrens, S. (2012) Abundance, distribution, and activity of Fe(II)‐oxidizing and Fe(III)‐reducing microorganisms in hypersaline sediments of Lake Kasin, southern Russia. Applied and Environmental Microbiology, 78, 4386–4399. 22504804 10.1128/AEM.07637-11 PMC3370536

[emi413149-bib-0019] Finneran, K.T. , Housewright, M.E. & Lovley, D.R. (2002) Multiple influences of nitrate on uranium solubility during bioremediation of uranium‐contaminated subsurface sediments. Environmental Microbiology, 4, 510–516. 12220407 10.1046/j.1462-2920.2002.00317.x

[emi413149-bib-0020] Gahan, C.S. , Sundkvist, J.E. , Dopson, M. & Sandström, Å. (2010) Effect of chloride on ferrous iron oxidation by a *Leptospirillum ferriphilum*‐dominated chemostat culture. Biotechnology and Bioengineering, 106, 422–431. 20198654 10.1002/bit.22709

[emi413149-bib-0021] Gu, B. , Schmitt, J. , Chen, Z. , Liang, L. & McCarthy, J.F. (1994) Adsorption and desorption of natural organic matter on iron oxide: mechanisms and models. Environmental Science & Technology, 28, 38–46. 22175831 10.1021/es00050a007

[emi413149-bib-0022] Guzman, M.S. , Rengasamy, K. , Binkley, M.M. , Jones, C. , Ranaivoarisoa, T.O. , Singh, R. et al. (2019) Phototrophic extracellular electron uptake is linked to carbon dioxide fixation in the bacterium Rhodopseudomonas palustris. Nature Communications, 10, 1355. 10.1038/s41467-019-09377-6 PMC6430793 30902976

[emi413149-bib-0023] Hafenbradl, D. , Keller, M. , Dirmeier, R. , Rachel, R. , Roßnagel, P. , Burggraf, S. et al. (1996) Ferroglobus placidus gen. nov., sp. nov., a novel hyperthermophilic archaeum that oxidizes Fe2+ at neutral pH under anoxic conditions. Archives of Microbiology, 166, 308–314. 8929276 10.1007/s002030050388

[emi413149-bib-0024] Han, X. , Tomaszewski, E.J. , Sorwat, J. , Pan, Y. , Kappler, A. & Byrne, J.M. (2020) Effect of microbial biomass and humic acids on abiotic and biotic magnetite formation. Environmental Science & Technology, 54, 4121–4130. 32129607 10.1021/acs.est.9b07095

[emi413149-bib-0025] Hansel, C.M. , Benner, S.G. & Fendorf, S. (2005) Competing Fe(II)‐induced mineralization pathways of ferrihydrite. Environmental Science & Technology, 39, 7147–7153. 16201641 10.1021/es050666z

[emi413149-bib-0026] Hansel, C.M. , Benner, S.G. , Neiss, J. , Dohnalkova, A. , Kukkadapu, R.K. & Fendorf, S. (2003) Secondary mineralization pathways induced by dissimilatory iron reduction of ferrihydrite under advective flow. Geochimica et Cosmochimica Acta, 67, 2977–2992.

[emi413149-bib-0027] Hansen, H.C.B. , Borggaard, O.K. & Sørensen, J. (1994) Evaluation of the free energy of formation of Fe(II)‐Fe(III) hydroxide‐sulphate (green rust) and its reduction of nitrite. Geochimica et Cosmochimica Acta, 58, 2599–2608.

[emi413149-bib-0028] He, S. , Tominski, C. , Kappler, A. , Behrens, S. & Roden, E.E. (2016) Metagenomic analyses of the autotrophic Fe(II)‐oxidizing, nitrate‐reducing enrichment culture KS. Applied and Environmental Microbiology, 82, 2656–2668. 26896135 10.1128/AEM.03493-15 PMC4836422

[emi413149-bib-0029] Hedrich, S. , Schlömann, M. & Johnson, D.B. (2011) The iron‐oxidizing proteobacteria. Microbiology, 157, 1551–1564. 21511765 10.1099/mic.0.045344-0

[emi413149-bib-0030] Hegler, F. , Posth, N.R. , Jiang, J. & Kappler, A. (2008) Physiology of phototrophic iron (II)‐oxidizing bacteria: implications for modern and ancient environments. FEMS Microbiology Ecology, 66, 250–260. 18811650 10.1111/j.1574-6941.2008.00592.x

[emi413149-bib-0031] Huang, Y.M. , Straub, D. , Blackwell, N. , Kappler, A. & Kleindienst, S. (2021) Meta‐omics reveal Gallionellaceae and Rhodanobacter species as interdependent key players for Fe(II) oxidation and nitrate reduction in the autotrophic enrichment culture KS. Applied and Environmental Microbiology, 87, e0049621. 34020935 10.1128/AEM.00496-21 PMC8276803

[emi413149-bib-0032] Huang, Y.M. , Straub, D. , Kappler, A. , Smith, N. , Blackwell, N. & Kleindienst, S. (2021) A novel enrichment culture highlights core features of microbial networks contributing to autotrophic Fe(II) oxidation coupled to nitrate reduction. Microbial Physiology, 31, 280–295. 34218232 10.1159/000517083

[emi413149-bib-0033] Jakus, N. , Blackwell, N. , Osenbrück, K. , Straub, D. , Byrne, J.M. , Wang, Z. et al. (2021) Nitrate removal by a novel lithoautotrophic nitrate‐reducing iron(II)‐oxidizing culture enriched from a pyrite‐rich limestone aquifer. Applied and Environmental Microbiology, 87, e00460‐21. 34085863 10.1128/AEM.00460-21 PMC8373248

[emi413149-bib-0034] Jakus, N. , Mellage, A. , Hoeschen, C. , Maisch, M. , Byrne, J.M. , Mueller, C.W. et al. (2021) Anaerobic neutrophilic pyrite oxidation by a chemolithoautotrophic nitrate‐reducing iron(II)‐oxidizing culture enriched from a fractured aquifer. Environmental Science & Technology, 55, 9876–9884. 34247483 10.1021/acs.est.1c02049

[emi413149-bib-0035] Jette, E.R. & Foote, F. (1933) An x‐ray study of the Wüstite (FeO) solid solutions. The Journal of Chemical Physics, 1, 29–36.

[emi413149-bib-0036] Kampschreur, M.J. , Kleerebezem, R. , de Vet, W.W. & van Loosdrecht, M.C. (2011) Reduced iron induced nitric oxide and nitrous oxide emission. Water Research, 45, 5945–5952. 21940030 10.1016/j.watres.2011.08.056

[emi413149-bib-0037] Kappler, A. , Becker, S. & Enright, A.M.L. (2021) Metals, microbes, and minerals—The biogeochemical side of life. In: Peter, K. & Martha Sosa, T. (Eds.) Living on iron, Vol. 21. Berlin: Walter de Gruyter GmbH & Co KG, pp. 185–228.

[emi413149-bib-0038] Kappler, A. , Bryce, C. , Mansor, M. , Lueder, U. , Byrne, J.M. & Swanner, E.D. (2021) An evolving view on biogeochemical cycling of iron. Nature Reviews Microbiology, 19, 360–374. 33526911 10.1038/s41579-020-00502-7

[emi413149-bib-0039] Kappler, A. , Schink, B. & Newman, D.K. (2005) Fe(III) mineral formation and cell encrustation by the nitrate‐dependent Fe(II)‐oxidizer strain BoFeN1. Geobiology, 3, 235–245.

[emi413149-bib-0040] Kendall, B. , Anbar, A.D. , Kappler, A. & Konhauser, K.O. (2012) The global iron cycle. Fundamentals of Geobiology, 1, 65–92.

[emi413149-bib-0041] Kim, H. , Kaown, D. , Mayer, B. , Lee, J.‐Y. , Hyun, Y. & Lee, K.‐K. (2015) Identifying the sources of nitrate contamination of groundwater in an agricultural area (Haean basin, Korea) using isotope and microbial community analyses. Science of the Total Environment, 533, 566–575. 26204420 10.1016/j.scitotenv.2015.06.080

[emi413149-bib-0042] Klueglein, N. & Kappler, A. (2013) Abiotic oxidation of Fe(II) by reactive nitrogen species in cultures of the nitrate‐reducing Fe(II) oxidizer Acidovorax sp. BoFeN1 ‐ questioning the existence of enzymatic Fe(II) oxidation. Geobiology, 11, 180–190. 23205609 10.1111/gbi.12019

[emi413149-bib-0043] Klueglein, N. , Zeitvogel, F. , Stierhof, Y.D. , Floetenmeyer, M. , Konhauser, K.O. , Kappler, A. et al. (2014) Potential role of nitrite for abiotic Fe(II) oxidation and cell encrustation during nitrate reduction by denitrifying bacteria. Applied and Environmental Microbiology, 80, 1051–1061. 24271182 10.1128/AEM.03277-13 PMC3911208

[emi413149-bib-0044] Laufer, K. , Byrne, J.M. , Glombitza, C. , Schmidt, C. , Jørgensen, B.B. & Kappler, A. (2016) Anaerobic microbial Fe(II) oxidation and Fe(III) reduction in coastal marine sediments controlled by organic carbon content. Environmental Microbiology, 18, 3159–3174. 27234371 10.1111/1462-2920.13387

[emi413149-bib-0045] Liu, T. , Chen, D. , Li, X. & Li, F. (2019) Microbially mediated coupling of nitrate reduction and Fe(II) oxidation under anoxic conditions. FEMS Microbiology Ecology, 95, fiz030. 30844067 10.1093/femsec/fiz030

[emi413149-bib-0046] Liu, T. , Chen, D. , Luo, X. , Li, X. & Li, F. (2019) Microbially mediated nitrate‐reducing Fe(II) oxidation: quantification of chemodenitrification and biological reactions. Geochimica et Cosmochimica Acta, 256, 97–115.

[emi413149-bib-0048] Melton, E.D. , Schmidt, C. & Kappler, A. (2012) Microbial iron(II) oxidation in littoral freshwater lake sediment: the potential for competition between phototrophic vs. nitrate‐reducing iron(II)‐oxidizers. Frontiers in Microbiology, 3, 197. 22666221 10.3389/fmicb.2012.00197 PMC3364526

[emi413149-bib-0049] Miot, J. , Benzerara, K. , Morin, G. , Bernard, S. , Beyssac, O. , Larquet, E. et al. (2009) Transformation of vivianite by anaerobic nitrate‐reducing iron‐oxidizing bacteria. Geobiology, 7, 373–384. 19573166 10.1111/j.1472-4669.2009.00203.x

[emi413149-bib-0050] Muehe, E.M. , Gerhardt, S. , Schink, B. & Kappler, A. (2009) Ecophysiology and the energetic benefit of mixotrophic Fe(II) oxidation by various strains of nitrate‐reducing bacteria. FEMS Microbiology Ecology, 70, 335–343. 19732145 10.1111/j.1574-6941.2009.00755.x

[emi413149-bib-0051] Nordhoff, M. , Tominski, C. , Halama, M. , Byrne, J.M. , Obst, M. , Kleindienst, S. et al. (2017) Insights into nitrate‐reducing Fe(II) oxidation mechanisms through analysis of cell‐mineral associations, cell encrustation, and mineralogy in the chemolithoautotrophic enrichment culture KS. Applied and Environmental Microbiology, 83, e00752–e00717. 28455336 10.1128/AEM.00752-17 PMC5478975

[emi413149-bib-0052] Ojumu, T.V. & Petersen, J. (2011) The kinetics of ferrous ion oxidation by Leptospirillum ferriphilum in continuous culture: the effect of pH. Hydrometallurgy, 106, 5–11.

[emi413149-bib-0053] Peiffer, S. , Kappler, A. , Haderlein, S.B. , Schmidt, C. , Byrne, J.M. , Kleindienst, S. et al. (2021) A biogeochemical–hydrological framework for the role of redox‐active compounds in aquatic systems. Nature Geoscience, 14, 264–272.

[emi413149-bib-0054] Roden, E.E. (2012) Microbial iron‐redox cycling in subsurface environments. Biochemical Society Transactions, 40, 1249–1256. 23176463 10.1042/BST20120202

[emi413149-bib-0056] Schmid, G. , Zeitvogel, F. , Hao, L. , Ingino, P. , Floetenmeyer, M. , Stierhof, Y.‐D. et al. (2014) 3‐D analysis of bacterial cell‐(iron)mineral aggregates formed during Fe(II) oxidation by the nitrate‐reducing Acidovorax sp. strain BoFeN1 using complementary microscopy tomography approaches. Geobiology, 12, 340–361. 24828365 10.1111/gbi.12088

[emi413149-bib-0057] Segre, C. , Leyarovska, N. , Chapman, L. , Lavender, W. , Plag, P. , King, A. et al. (2000) The MRCAT insertion device beamline at the advanced photon source. In: AIP conference proceedings, Vol. 521, no. 1. College Park, MD: American Institute of Physics, pp. 419–422.

[emi413149-bib-0059] Sørensen, J. & Thorling, L. (1991) Stimulation by lepidocrocite (7‐FeOOH) of Fe(II)‐dependent nitrite reduction. Geochimica et Cosmochimica Acta, 55, 1289–1294.

[emi413149-bib-0060] Stookey, L.L. (1970) Ferrozine ‐ a new spectrophotometric reagent for iron. Analytical Chemistry, 42, 779–781.

[emi413149-bib-0061] Straub, K.L. , Benz, M. , Schink, B. & Widdel, F. (1996) Anaerobic, nitrate‐dependent microbial oxidation of ferrous iron. Applied and Environmental Microbiology, 62, 1458–1460. 16535298 10.1128/aem.62.4.1458-1460.1996 PMC1388836

[emi413149-bib-0062] Straub, K.L. , Hanzlik, M. & Buchholz‐Cleven, B.E. (1998) The use of biologically produced ferrihydrite for the isolation of novel iron‐reducing bacteria. Systematic and Applied Microbiology, 21, 442–449. 9779609 10.1016/S0723-2020(98)80054-4

[emi413149-bib-0063] Swanner, E.D. , Mloszewska, A.M. , Cirpka, O.A. , Schoenberg, R. , Konhauser, K.O. & Kappler, A. (2015) Modulation of oxygen production in Archaean oceans by episodes of Fe(II) toxicity. Nature Geoscience, 8, 126–130.

[emi413149-bib-0064] Tiedje, J.M. (1988) Ecology of denitrification and dissimilatory nitrate reduction to ammonium. Biology of Anaerobic Microorganisms, 717, 179–244.

[emi413149-bib-0065] Tomaszewski, E.J. , Lee, S. , Rudolph, J. , Xu, H. & Ginder‐Vogel, M. (2017) The reactivity of Fe(II) associated with goethite formed during short redox cycles toward Cr(VI) reduction under oxic conditions. Chemical Geology, 464, 101–109.

[emi413149-bib-0066] Tominski, C. , Heyer, H. , Lösekann‐Behrens, T. , Behrens, S. & Kappler, A. (2018) Growth and population dynamics of the anaerobic Fe(II)‐oxidizing and nitrate‐reducing enrichment culture KS. Applied and Environmental Microbiology, 84, e02173–e02117. 29500257 10.1128/AEM.02173-17 PMC5930324

[emi413149-bib-0067] Visser, A.‐N. , Lehmann, M.F. , Rügner, H. , D'Affonseca, F.M. , Grathwohl, P. , Blackwell, N. et al. (2021) Fate of nitrate during groundwater recharge in a fractured karst aquifer in Southwest Germany. Hydrogeology Journal, 29, 1153–1171.

[emi413149-bib-0068] Ward, M.H. , Jones, R.R. , Brender, J.D. , De Kok, T.M. , Weyer, P.J. , Nolan, B.T. et al. (2018) Drinking water nitrate and human health: an updated review. International Journal of Environmental Research and Public Health, 15, 1557. 30041450 10.3390/ijerph15071557 PMC6068531

[emi413149-bib-0369] Webb, S.M. (2005) SIXpack: a graphical user interface for XAS analysis using IFEFFIT. Physica Scripta, T115, 1011.

[emi413149-bib-0069] Weber, K.A. , Urrutia, M.M. , Churchill, P.F. , Kukkadapu, R.K. & Roden, E.E. (2006) Anaerobic redox cycling of iron by freshwater sediment microorganisms. Environmental Microbiology, 8, 100–113. 16343326 10.1111/j.1462-2920.2005.00873.x

[emi413149-bib-0070] Weusthuis, R.A. , Pronk, J.T. , Van Den Broek, P. & Van Dijken, J. (1994) Chemostat cultivation as a tool for studies on sugar transport in yeasts. Microbiological Reviews, 58, 616–630. 7854249 10.1128/mr.58.4.616-630.1994 PMC372984

[emi413149-bib-0071] Widdel, F. , Schnell, S. , Heising, S. , Ehrenreich, A. , Assmus, B. & Schink, B. (1993) Ferrous iron oxidation by anoxygenic phototrophic bacteria. Nature, 362, 834–836.

