# Supplementary material for: Continuous cultivation of the lithoautotrophic nitrate‐reducing Fe(II)‐oxidizing culture KS in a chemostat bioreactor
Source: Environ Microbiol Rep. 2023 Mar 29;15(4):324–34. doi: 10.1111/1758-2229.13149 (PMC10316368; doi:10.1111/1758-2229.13149)
Supplement: Supplementary file 2 — Data S2. Supporting Information. [file EMI4-15-324-s002.pdf]

Continuous cultivation of the lithoautotrophic nitrate-reducing Fe(II)-oxidizing culture KS in a chemostat bioreactor

T. Bayer[1], E.J. Tomaszewski[1,†], C. Bryce[2], A. Kappler[1,3] and J.M. Byrne[2,]*

[1]Geomicrobiology Group, Center for Applied Geoscience, University of Tuebingen, Schnarrenbergstraße 94-96, 72076 Tuebingen, Germany.

[†] **Current address:** U.S. Geological Survey, 3215 Marine St. Boulder, CO 80303 USA.

[2]School of Earth Sciences, University of Bristol, Queens Road BS8 1RJ, Bristol, United Kingdom.

[3]Cluster of Excellence: EXC 2124: Controlling Microbes to Fight Infection, Tuebingen, Germany.

***Correspondence:**
James M. Byrne
james.byrne@bristol.ac.uk

Supplementary information

Chemostat setup

The chemostat used in this study (see reaction vessel in Fig. 1) was model BioFlo®/CelliGen®115, Benchtop Fermentor & Bioreactor (New Brunswick, an Eppendorf company). Manual No: M1369-0050, Revision B, June 2, 2009 (New Brunswick Scientific, Edison, USA; software BF115 Rev B). It consists of a reaction chamber and a control unit. All metal surfaces are 316L or 316 stainless steel. Separate parts included:

1) The reaction vessel, made from borosilicate glass, total volume of approximately 1 L. The reaction chamber is sealed airtight with a stainless-steel head plate with ten openings. The reaction chamber is tightly closed with a rubber seal. The reaction chamber can be enveloped in a Peltier-element-type heating vest, for maintaining a constant temperature. The chamber is attached to a four-footed stainless-steel holder.
2) The control unit, equipped with a touchscreen and various connection ports, allows following and adjusting pH, dissolved oxygen concentration (DO), temperature (T), water level and agitation.

Unless denoted otherwise, all connections for gas and medium exchange consist of stainless-steel capillaries (I/16” outer diameter, 1.0 mm inner diameter, ref. number 7.590133 CH.211661, Ziemer Chromatography; Klaus Ziemer GmbH, Langerwehe, Germany). Two types of tubing were used to enable pumping with peristaltic pumps. For external pumps, black pumping tubing (ID: 0.74 mm, wall: 0.91 mm, reference 070652 – 07i / SC0257, IDEX, Health & Science, ISMATEC®, Wertheim, Germany) was used. For the built-in pumps, yellow pumping tubing were used (Flexible Plastic Tubing, ID: 0.8 mm, OD: 4.0 mm, wall: 1.6 mm, PharMed® - BPT, Saint-Gobain Performance Plastics, Charny, France). External pumps were set to

continuously pump medium in and out of the reaction vessel. Pumping in rate (15 ml h^{-1}) was greater than pumping out (8 ml h^{-1}) to ensure constant volume. Using the controller units' *loops* option an additional pump was set to start at approximately 710 ml and reduced the volume back to 700 ml.

All sensors were attached using mounting threads, sealed with a Teflon and rubber ring, and additionally sealed with a Teflon thin foil (Maagtechnic, 60 g/m^2, BAM Tgb. EN751-3 FRp) on the inside of all threads. All other ports were sealed using rubber stoppers. The system was continuously flushed with an overpressure of about 10 mbar N_2/CO_2 (90:10, v/v). N_2/CO_2 was supplied from an external gas bottle (UN 1956, verdichtetes Gas, N.A.G., Westfalen AG, Münster, Germany. To maintain sterile conditions, the gas was fed through a cotton filled glass syringe, that was previously oven-sterilized (180°C, 4.5 h). This glass syringe was connected to a gas splitter made of stainless steel with a *Luer-lock* system. All air flow was led to a Schott bottle filled with sterilized, anoxic water. Here, all pressure will accumulate and gradually escape. To ensure that there is an actual gas flow, bubbles of outgassing air could be seen in the bottle ("*pressure valve*").

To enable continuous supply of bacterial growth medium to the system, Schott bottles with a volume of 2 L were chosen. Gas entered the medium bottle from the gas-splitter and then led to i) the chemostat and ii) the waste bottle.

After sterilization of the chemostats' reaction chamber and attaching the gas flow, the mixing motor was set to 200 rpm to purge oxygen from the system. The sterilized, polarized, and calibrated dissolved oxygen (DO) sensor was attached.

A 2 L Schott bottle was connected for outflow collection before autoclaving and was not removed unless it was filled up. In that case, the bottle was replaced with another sterilized,

degassed 2 L Schott bottle. Gas flow to this waste bottle was connected from the medium supply and the reaction vessel. Pumping from top and bottom layers of the reaction vessel were also fed into this bottle. A single capillary was connected to the previously mentioned *pressure-valve* bottle.

Chemicals and materials

All chemicals were at least of analytical grade. The water used was ultra-pure (Milli-Q, A10, Merck-Millipore, Billerica, USA). Anoxic solutions were prepared by either purging with N_2 or N_2/CO_2 and stored in glass containers sealed with butyl stoppers. All utensils and glassware were sterilized by autoclaving (121°C for 20 min) or by baking in an oven (180°C for 4.5 h).

Detailed sampling descriptions

Geochemical analyses

Samples from the chemostat were transferred with syringe and needle into a sterile, into an anoxic and sterile glass vial and brought into a glovebox (100% N_2, MBraun Germany). Batch experiments were sampled in the glovebox. Under anoxic conditions, 1 ml was pipetted into an Eppendorf tube and centrifuged (minispin, Eppendorf) for 10 minutes at 13.4k rpm. The sample was split into the supernatant and pellet for aqueous and solid phase Fe (Fe_{aq} and Fe_s respectively), NO_3^-, and NO_2^- analyses. For Fe_{aq}, supernatant was 10x diluted in 40 mM sulfamic acid in 1 M hydrochloric acid (1M HCl_{SA}) to prevent Fe(II) oxidation by RNS (Klueglein et al., 2014; Schaedler et al., 2017). For NO_3^-, supernatant was 20x diluted in anoxic Milli-Q. For NO_2^-, 750 µl were separated into a new Eppendorf tube. All samples were stored anoxically at 4°C until measurement. The pellet was dissolved for 1 h in 1 ml of anoxic 6 M

HCl_{SA} for Fe_s measurements. Fe(II) and total Fe (Fe(T)) were determined using the spectrophotometric ferrozine assay as described by Stookey (1970) but adapted for microtiter plates (96 well assay Plate, COStar, Kennebunk, USA). Absorbance was measured at 562 nm using a plate reader (Multiskan^{TM} GO Microplate Spectrophotometer, Thermo Scientific). NO_3^- and NO_2^- were measured using a continuous-flow analyser (Seal Analytical; Norderstedt, Germany) with a dialysis membrane for iron removal. Here, NO_3^- is reduced to NO_2^- with a solution of hydrazine sulphate, and then concentrations are determined photometrically with N-1-naphtyethylendiamin at a wavelength of 520 nm.

Mössbauer spectroscopy

For Mössbauer spectroscopy, 8 ml of sample was transferred to the glovebox. Minerals were collected by filtration through a 0.45 µm pore-size syringe filter (Millipore membrane). The filter was then embedded between two layers of Kapton tape foil and stored frozen (-20°C) and anoxically until analysis. Samples were inserted into a closed-cycle exchange gas cryostat (SHI-650-5; Janis Research, USA). Spectra were collected at 77 K using a constant acceleration drive system (WissEl, Blieskastel, Germany). Gamma radiation was emitted by a ^{57}Co-source embedded in a rhodium matrix. Sample spectra were calibrated against a 7-µm-thick Fe(0) foil at room temperature. The Recoil software (University of Ottawa, Canada) was used for fitting spectra using the Voigt-based fitting model. The Lorentzian half-width-half-maximum (HWHM) value was kept constant at 0.133 mm/s. The sample spectra were analysed with respect to the isomer shift (δ) values and the quadrupole splitting (ΔE_Q) and the Gaussian width (standard deviation) of the ΔE_Q was used to account for line broadening until the fit was reasonable. For µ-XRD, samples were collected and air dried in an Eppendorf tube in an oven at 27°C inside an anoxic glovebox.

μ-XRD

μ-XRD was performed on the dried material using Bruker's D8 Discover GADDS XRD2 micro-diffractometer equipped with a standard sealed tube with Co-cathode (Co Kα radiation, λ = 0.154 nm; 30 kV/30 mA). The total measurement time was 240 s at two detector positions, 15° and 40°. Phase identification was validated using Match! software version 3.6.2.121 with Crystallography Open Database (COD-Inorg REV211633 2018.19.25).

X-ray adsorption

For X-ray adsorption spectroscopy (XAS), an anoxically dried sample taken after 40 days (no geochemistry measured) was diluted with polyvinylpyrrolidone and pressed into 7-mm pellets using a KBr pellet press (International Crystal). The pellet was anoxically sealed in Kapton tape. X-ray absorption spectroscopy was performed at the Advanced Photon Source (APS) Materials Research Collaborative Access Team (MRCAT) beamline 10-ID-B at Argonne National Laboratory (Segre et al., 2000). Beamline 10-ID-B employs an undulator magnet source and a Si(111) monochromator. Spectra were collected at the Fe K-edge (7.112 keV) to k=0-16 $Å^{-1}$. Data reduction, normalization and calibration were performed using the ATHENA program in the Demeter software package (Ravel and Newville, 2005). The spectrum was calibrated to a Fe reference foil spectrum collected during data collection. Linear combination fitting (LCF) of k^3 weighted Fe EXAFS spectra was performed from k=3-12 $Å^{-1}$ using ferrihydrite, siderite, goethite, and lepidocrocite spectra collected at the same beamline. Other standards considered during fitting include a ferrihydrite-humic acid co-precipitate, a Fe(II)-natural organic matter co-precipitate and a Fe(III)-citrate complex, all of which were collected previously at various beamlines (Shimizu et al., 2013; Daugherty et al., 2017). This type of

fitting offers a semi-quantitative approach to understand the contribution of various Fe phases. SIXpack (Webb, 2005) software was used to perform LCF analysis.

SEM

Samples for scanning electron microscopy were fixed in 2.5% glutaraldehyde overnight at 4°C. After washing three times with DI-water, samples were applied to a Poly-L-Lysine coated glass slide. Dehydration was performed on the glass slides by stepwise water replacement with increasing concentrations of pure ethanol (30, 50, 70, 90, and 2x 100 %), followed by washing in hexamethyldisilazane for 2x 30 seconds. Samples were then mounted onto SEM aluminium stubs using double sided spectra-carbon tape (Plano, Germany) and sputter coated with a 12 nm platinum layer (Baltec SCD005 sputter-coater). Micrographs were collected using a JEOL JSM-6500F field emission SEM with a Schottky-field-emitter at a working distance of approximately 10 mm at the Centre for Light-Matter Interaction, Sensor & Analytics ($LISA^+$), University of Tuebingen.

Figure S1: Overview of the chemostat.

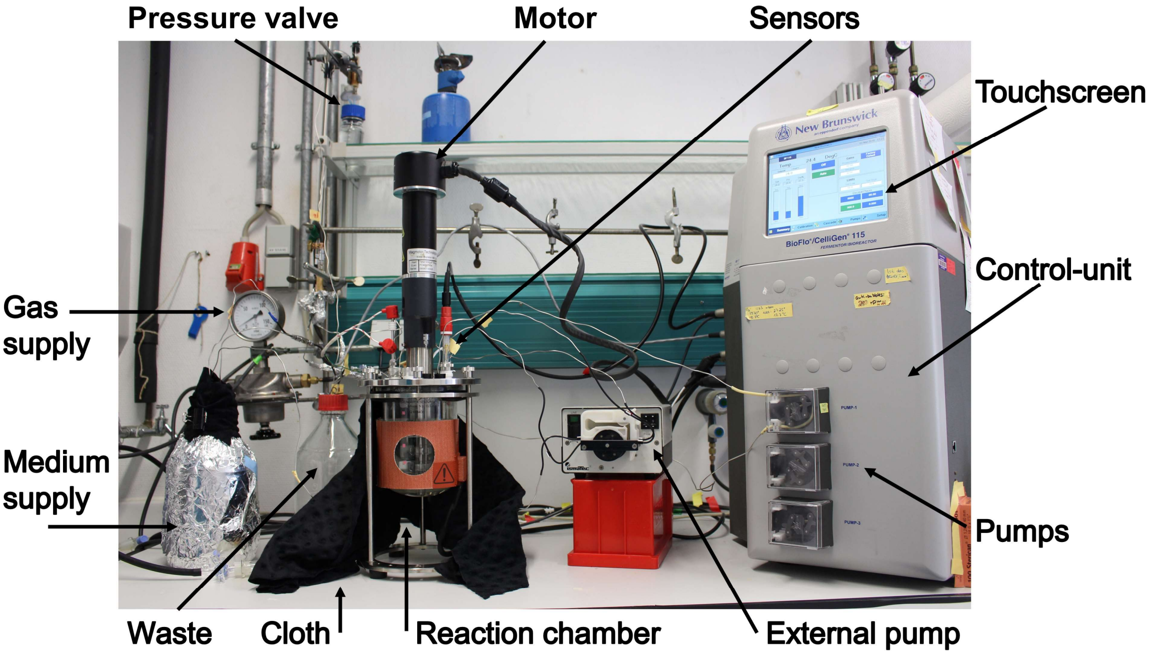

Figure S2: Logged data collected for temperature, pH and DO (dissolved oxygen) over time in the chemostat reaction chamber.

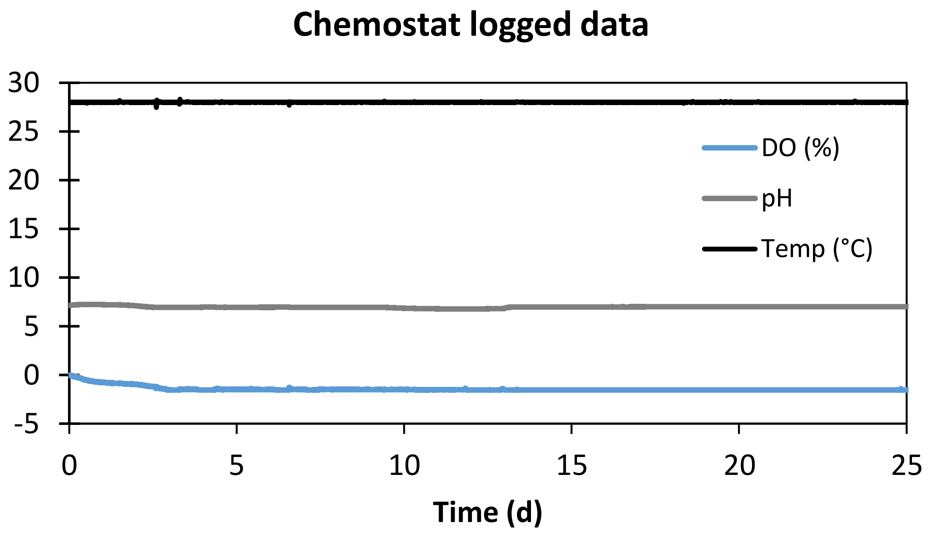

Figure S3: Fe(II) oxidation in the chemostat. Oxidation rates were calculated by first measuring the Fe(II) concentration $[Fe(II)_{meas}]_{t(0\text{ and }1)}$ in the reactor for the first two timepoints, where no pumping was turned on yet. Then, to account for mixing (dilution) effects, the expected Fe(II) concentration ($[Fe(II)]_{expected,t(x+1)}$; i.e. what we would be expected if there was no microbial Fe(II) oxidation and only addition and mixing of Fe(II) from the supplied medium (supplied: 2.82 mM $Fe(II)_{aq}$ and 7.38 mM $Fe(II)_{s}$) was calculated. The difference between $[Fe(II)_{meas}]_{t(x+1)}$ (with x ≥ 1) and $[Fe(II)]_{expected,\ t(x+1)}$ thus indicated how much Fe(II) was oxidized microbially over a given time period.

The rate of microbial oxidation of Fe(II) in the chemostat was therefore calculated according to equations 1 and 2.

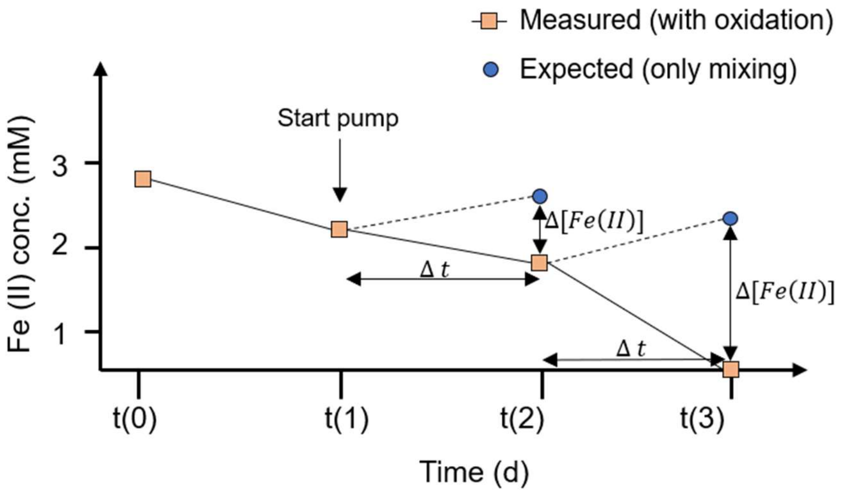

$$\frac{d[Fe(II)]}{dt} = \frac{[Fe(II)_{expected}]_{t(x+1)} - [Fe(II)_{meas}]_{t(x+1)}}{\Delta t} \qquad \text{eq. 1}$$

Where Δt is the difference in time between two measured points t(x+1) and t(x).

$$[Fe(II)_{expected}]_{t(x+1)} = [Fe(II)_{meas}]_{t(x)} \frac{(V_{chem} - (R_{addition}\Delta t))}{V_{chem}} + [Fe(II)_{sup}] \frac{R_{addition}\Delta t}{V_{chem}} \qquad \text{eq. 2}$$

Where V_{chem} is the volume of the chemostat (700 ml); $R_{addition}$ is the flow rate of medium supplied to the chemostat (15 ml h^{-1}); $[Fe(II)_{sup}]$ is the concentration of Fe(II) added into the chemostat by pumping (2.82 mM $Fe(II)_{aq}$ and 7.38 mM $Fe(II)_{s}$ respectively. Note that x ≥ 1 (since pumping did not start until after t(1)).

The equation for the expected concentration $[Fe(II)_{expected}]_{t(x+1)}$ accounts for the mixing of Fe(II) already present in the chemostat $[Fe(II)_{meas}]_{t(x)}$ by medium supplied to the chemostat $[Fe(II)_{sup}]$ (constant). Medium was supplied at a pumping rate of 15 ml h^{-1}. Every

24 h, 360 ml of $[Fe(II)_{sup}]$ was added to 340 ml of $[Fe(II)_{meas}]_{t(x)}$, which remained from the previous time point, reaching a total volume of 700 ml in the chemostat. The difference between the measured concentration $[Fe(II)_{meas}]_{t(x+1)}$ and the expected concentration $[Fe(II)_{expected}]_{t(x+1)}$ corresponds to the amount of Fe(II) oxidized between t(x) to t(x+1). Calculations were performed separately for $Fe(II)_{aq}$ and $Fe(II)_{s}$.

Figure S4: Batch experiments performed as control: small volume (25 ml), shaken at 50 rpm. Fe(II) (circles) and Fe(III) (squares) measured in aqueous (grey) and solid phase (orange) during cultivation of culture KS with 10 mM Fe(II) and 4 mM of NO_3^- in three bottles A, B, and C which represent biological replicates. D: abiotic control.

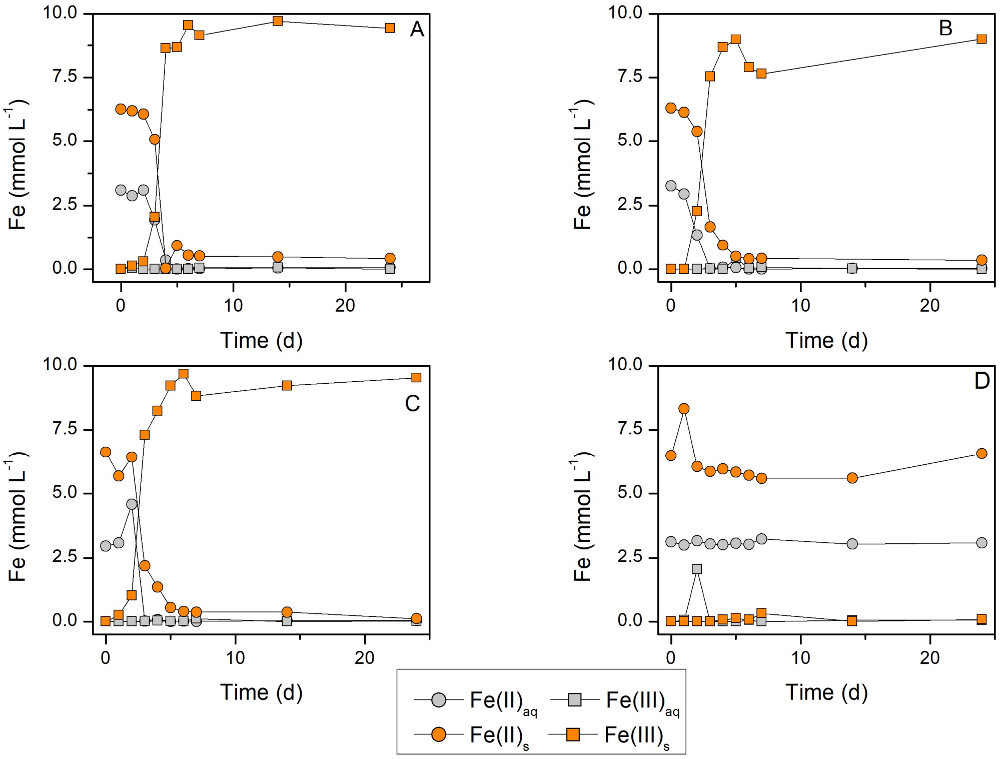

Figure S5: Batch experiments performed as control: small volume (25 ml), static. Fe(II) (circles) and Fe(III) (squares) measured in aqueous (grey) and solid phase (orange) during cultivation of culture KS with 10 mM Fe(II) and 4 mM of NO_3^- in three bottles A, B, and C which represent biological replicates. D: abiotic control.

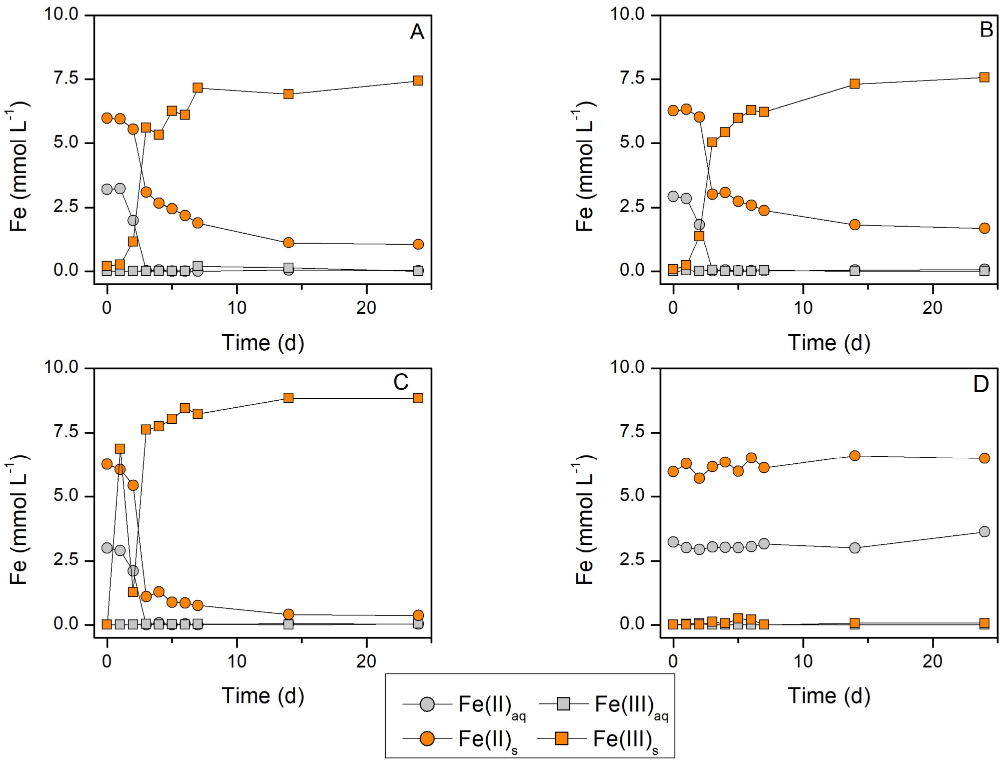

Figure S6: Batch experiments performed as control: big volume (700 ml), shaken at 50 rpm. Fe(II) (circles) and Fe(III) (squares) measured in aqueous (grey) and solid phase (orange) during cultivation of culture KS with 10 mM Fe(II) and 4 mM of NO_3^- in three bottles A, B, and C which represent biological replicates. D: abiotic control.

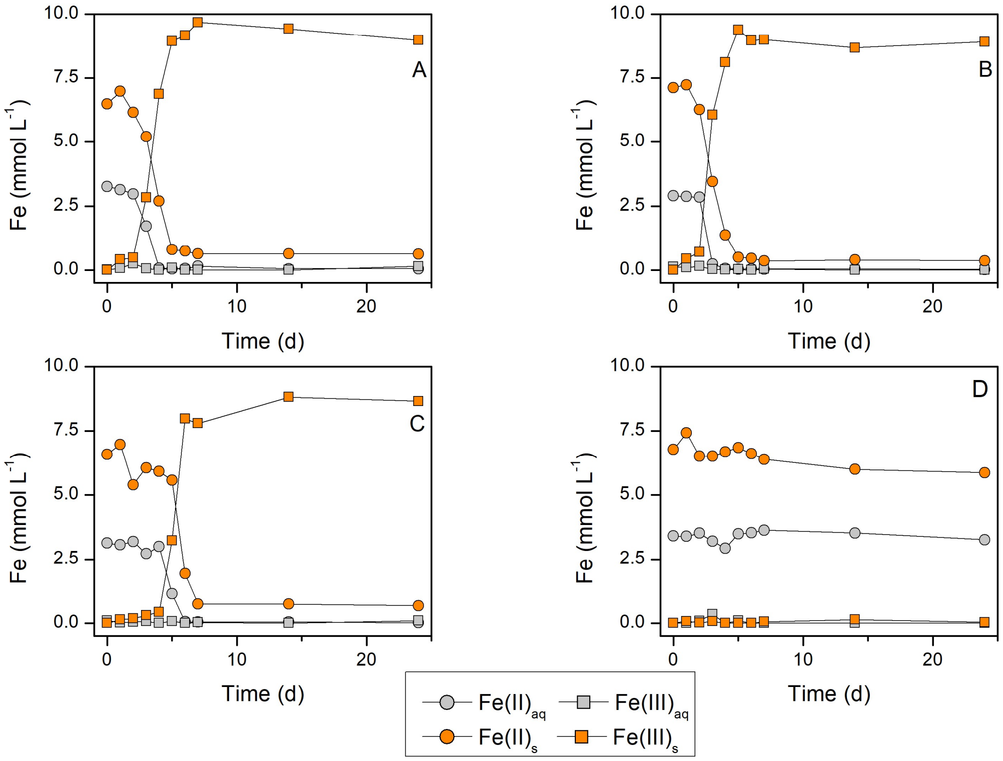

Figure S7: Batch experiments performed as control: big volume (700 ml), static. Fe(II) (circles) and Fe(III) (squares) measured in aqueous (grey) and solid phase (orange) during cultivation of culture KS with 10 mM Fe(II) and 4 mM of NO_3^- in three bottles A, B, and C which represent biological replicates. D: abiotic control.

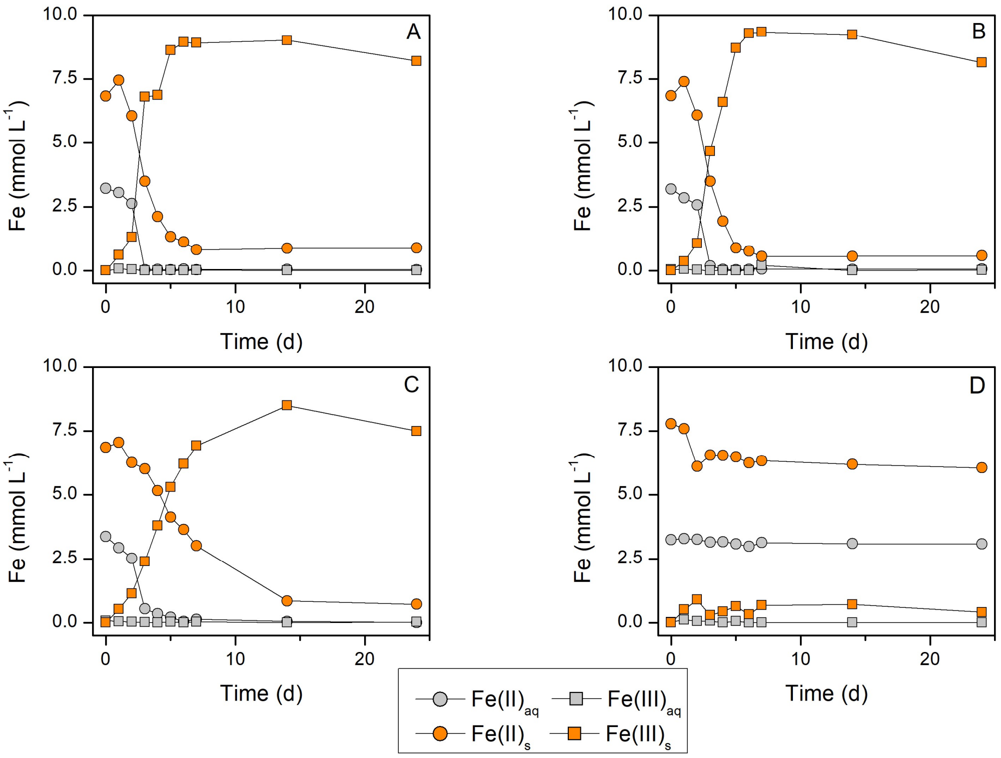

Figure S8: Batch experiments performed as control: small volume (25 ml), shaken at 50 rpm. NO_3^- (black circles) and NO_2^- (blue circles) measured in aqueous phase during cultivation of culture KS with 10 mM Fe(II) and 4 mM of NO_3^- in three bottles A, B, and C which represent biological replicates. D: abiotic control. **Note differences in y-axes scale to better show the variance in the biological data.**

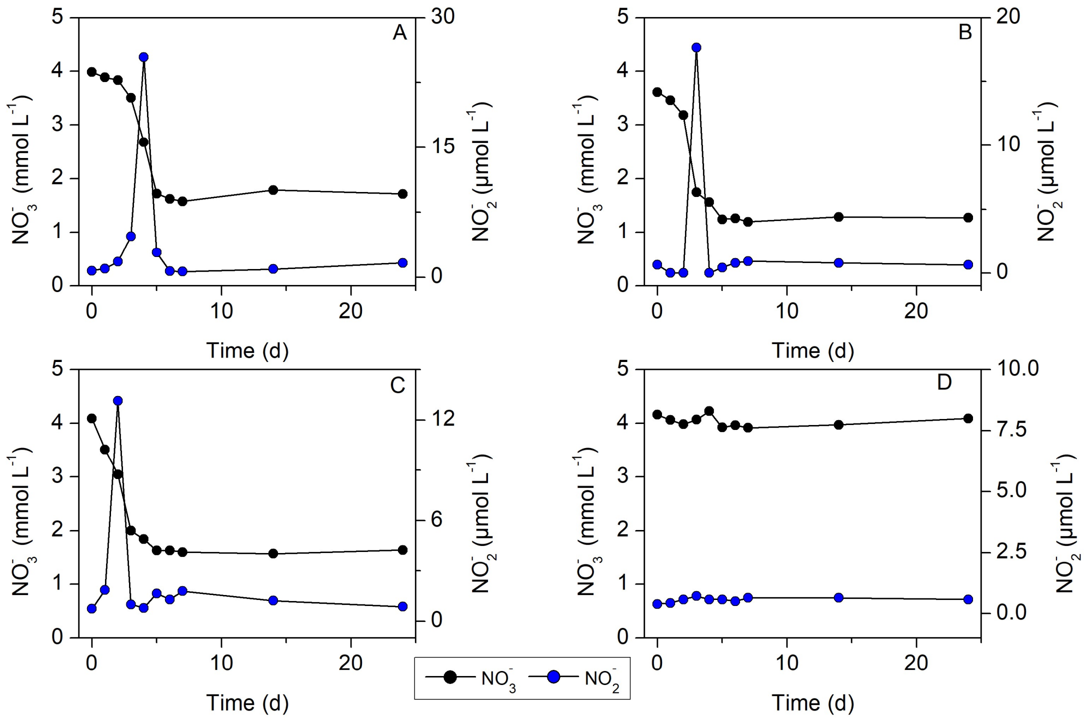

Figure S9: Batch experiments performed as control: small volume (25 ml), static. NO_3^- (black circles) and NO_2^- (blue circles) measured in aqueous phase during cultivation of culture KS with 10 mM Fe(II) and 4 mM of NO_3^- in three bottles A, B, and C which represent biological replicates. D: abiotic control. **Note differences in y-axes scale to better show the variance in the biological data.**

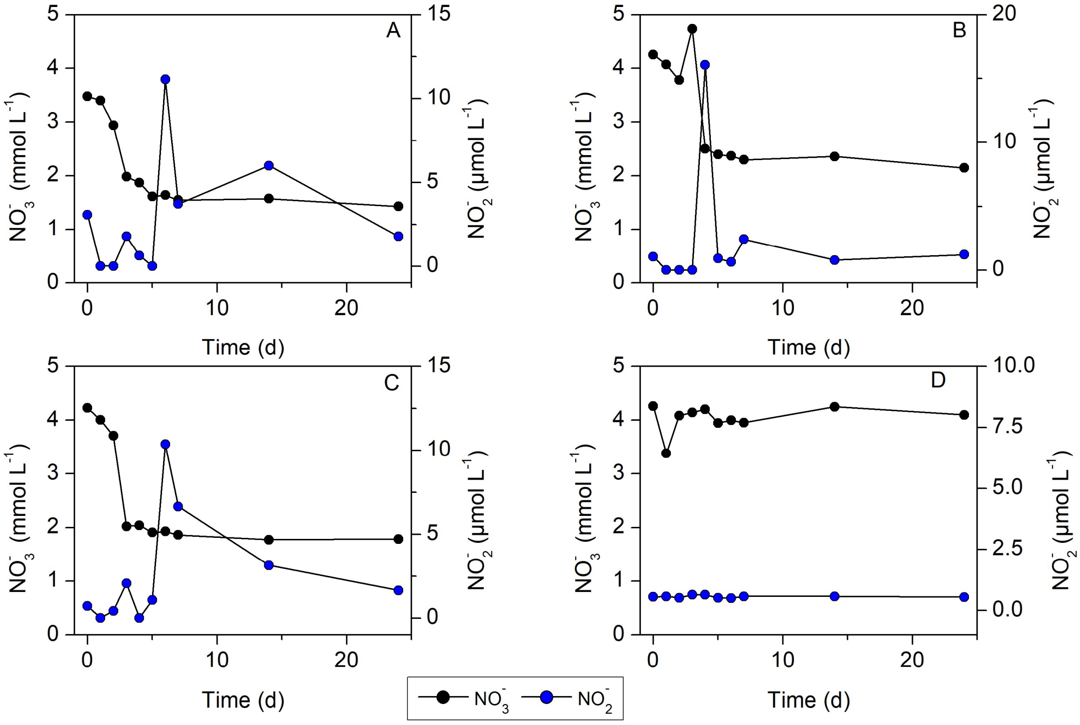

Figure S10: Batch experiments performed as control: big volume (700 ml), shaken at 50 rpm. NO_3^- (black circles) and NO_2^- (blue circles) measured in aqueous phase during cultivation of culture KS with 10 mM Fe(II) and 4 mM of NO_3^- in three bottles A, B, and C which represent biological replicates. D: abiotic control. **Note differences in y-axes scale to better show the variance in the biological data.**

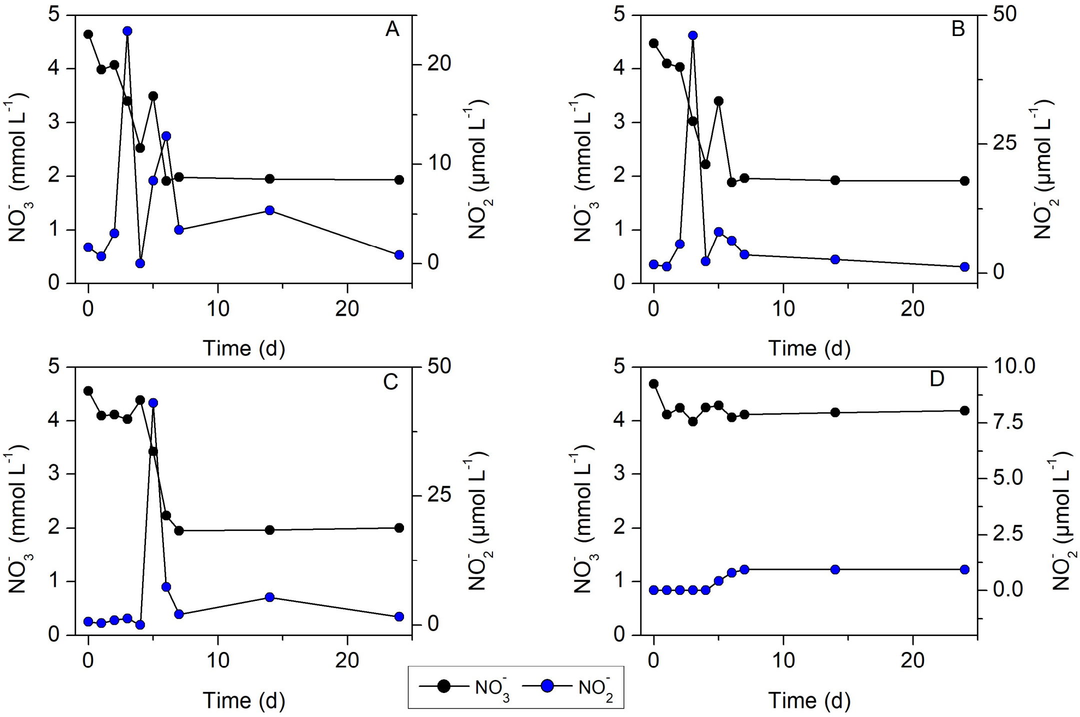

Figure S11: Batch experiments performed as control: big volume (700 ml), static. NO_3^- (black circles) and NO_2^- (blue circles) measured in aqueous phase during cultivation of culture KS with 10 mM Fe(II) and 4 mM of NO_3^- in three bottles A, B, and C which represent biological replicates. D: abiotic control. **Note differences in y-axes scale to better show the variance in the biological data.**

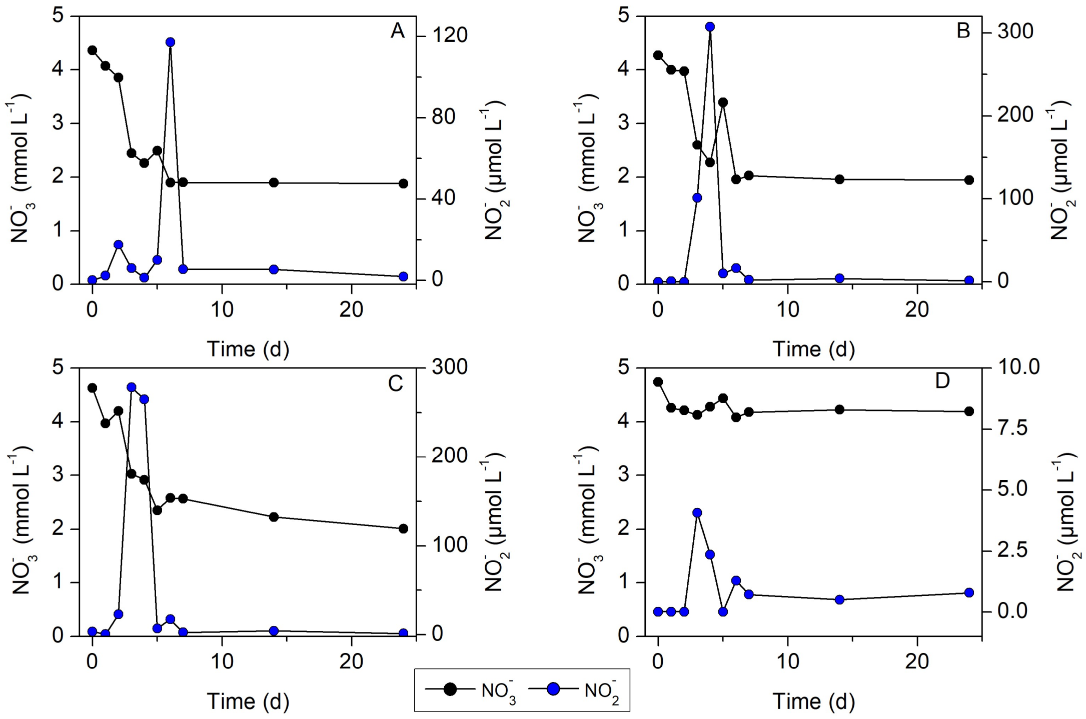

Figure S12. µ-XRD patterns for collected and oven dried minerals precipitates from) 25 ml and 700 ml batch experiments (both shaken) and the chemostat for timepoint 7 (c) *chem1*) and at 24 days (d), *chem2*) of autotrophically growing culture KS with 10 mM Fe(II) and 4 mM of nitrate. Noise at 2-Theta of approx. 51° and 65° is due to the sample holder (Si-wafer). Ha: halite, Ho: sample holder, W: wuestite, V: vivianite.

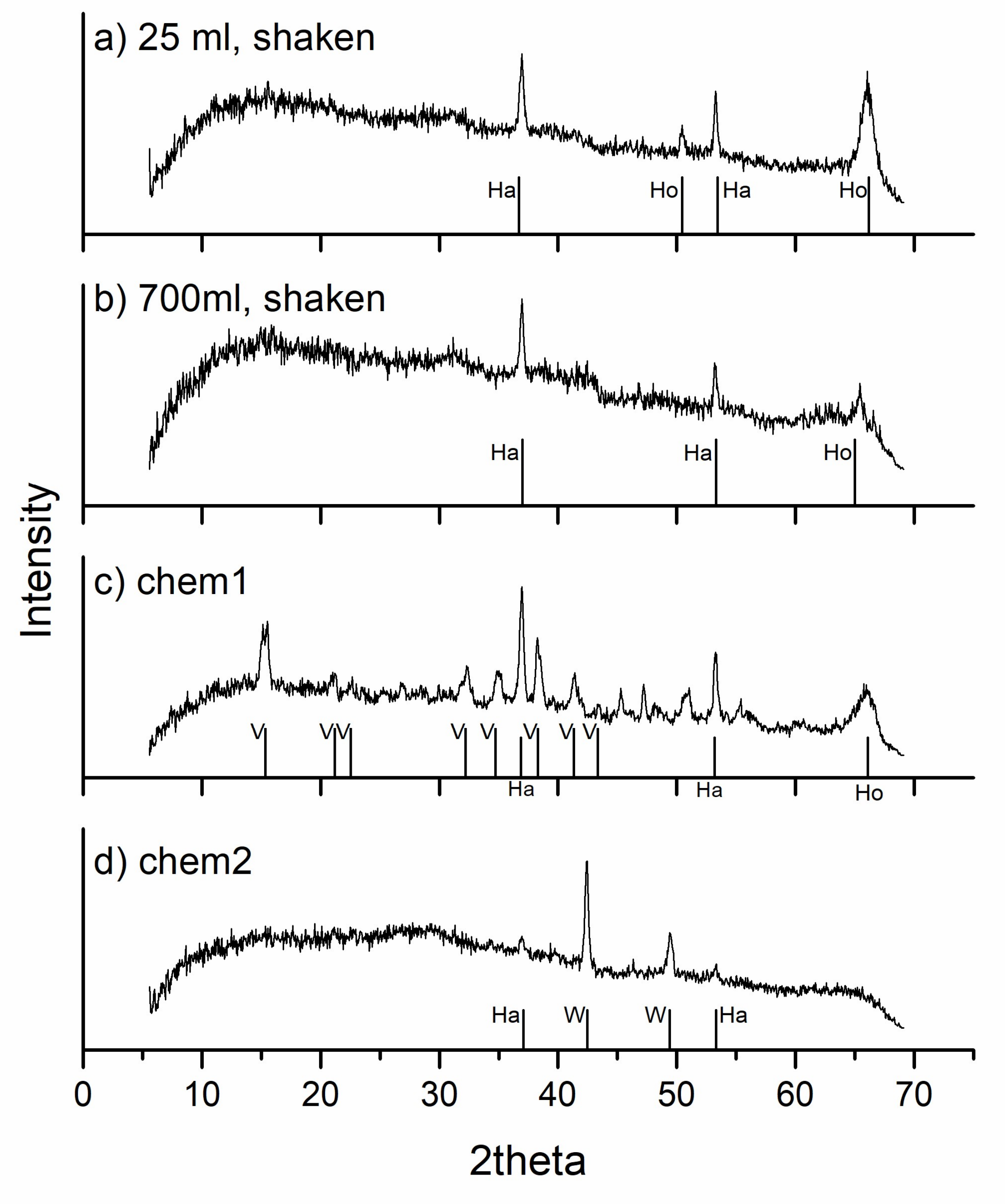

Figure S13. X-ray absorption spectroscopy analysis of collected mineral precipitate collected from the very bottom of the chemostat bioreactor, after continuous cultivation of autotrophically grown culture KS for 40 days with 10 mM Fe(II) and 4 mM of nitrate. Recorded values and fitted data are displayed in solid and dashed lines respectively.

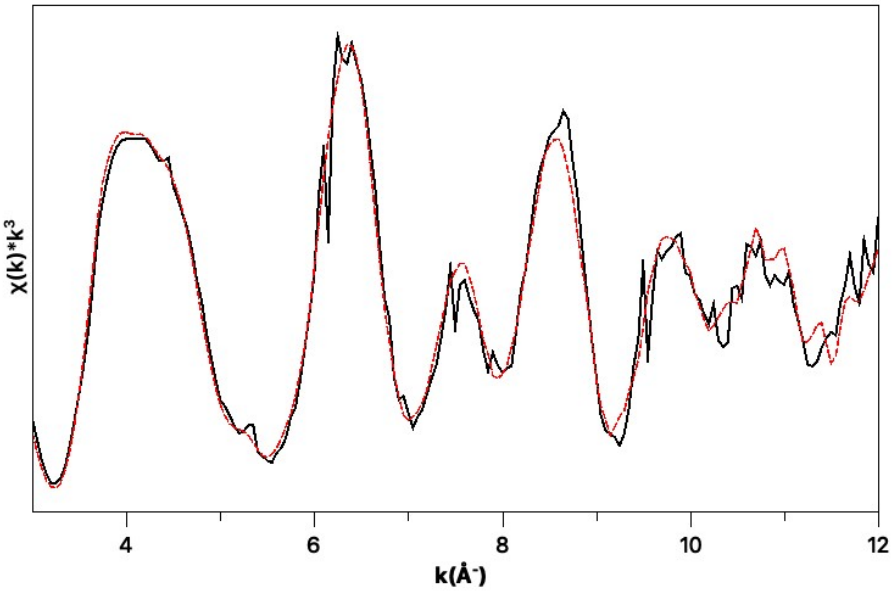

Figure S14. Composition of collected mineral precipitate sample from the very bottom of the chemostat bioreactor (analysed with X-ray absorption spectroscopy), after continuous cultivation of culture KS grown autotrophically for 40 days with 10 mM Fe(II) and 4 mM of nitrate.

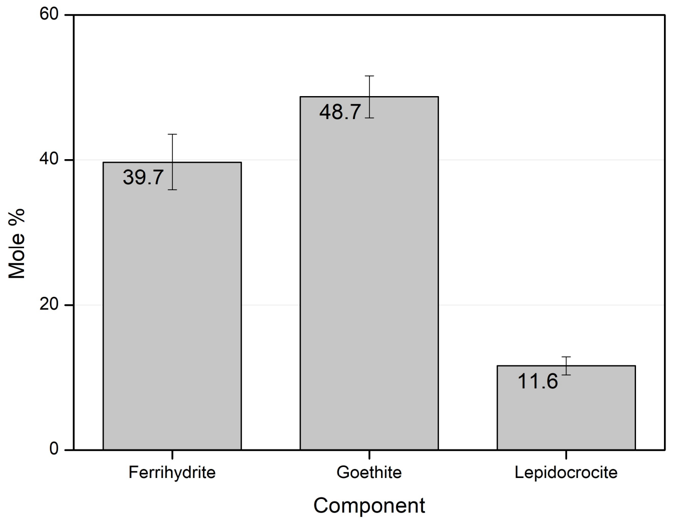

Figure S15. Relative amount of not, partially, or completely encrusted cells investigated and counted with scanning electron microscopy from continuous cultivation of culture KS under autotrophic conditions in the chemostat. A total of 78 cells were counted from the chemostat.

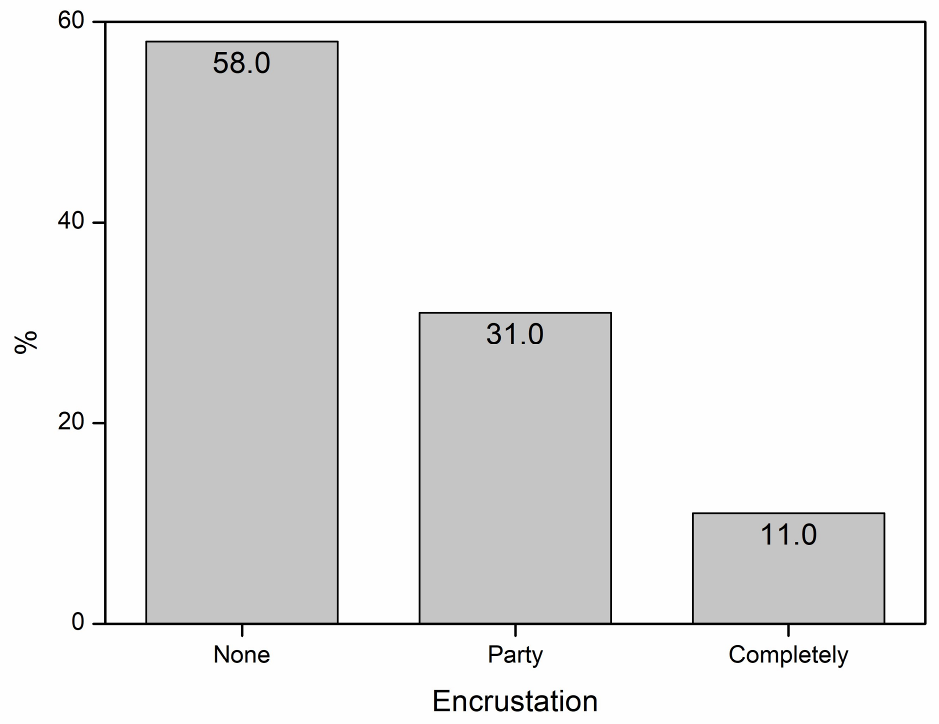

Figure S16. Fluorescence microscopy images of culture KS grown autotrophically with 10 mM Fe(II) and 4 mM of nitrate in the chemostat at day 24. Green colours represent live cells while magenta colours show dead cells. Cells were stained with D/L stain.

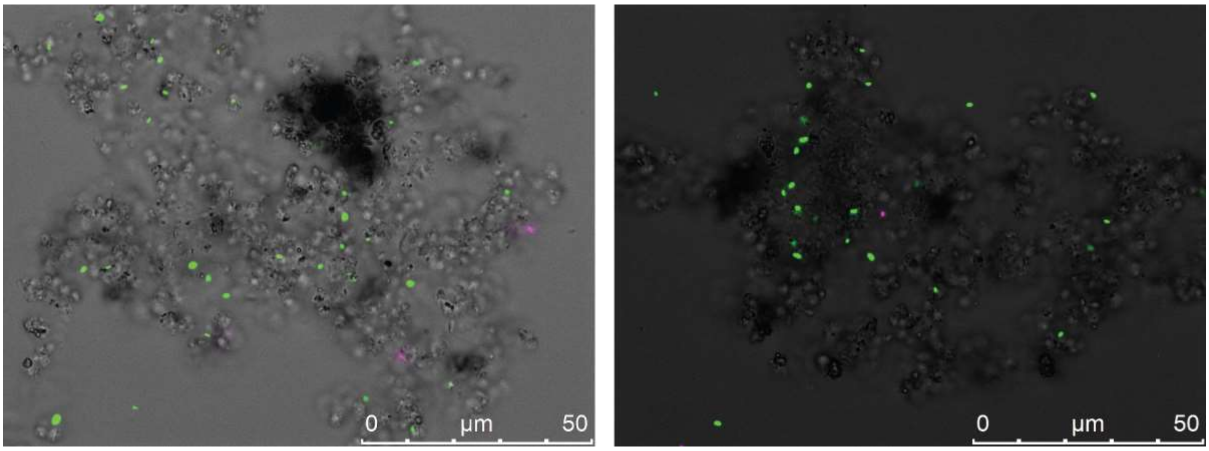

Previous run of the chemostat

A run conducted ahead of time of the research presented in the main manuscript is shown in figure S17.Here we can see that the lag phase of culture KS was much longer (around 10 days), this was caused by inoculating the culture after only one previous transfer instead of two. Overall, the data are fluctuating more as the pumping and sampling were not perfectly established yet. We see that a steep decrease of Fe(II) as soon as the oxidation of culture KS started. We confirm the biotic activity of culture KS with the concurrent decrease of nitrate in the systems. Oxidation rates for this run (as seen in table S3) show the same trend as for the experiment presented in the main manuscript: the rate for solid phase Fe(II) is greater than the rate for aqueous Fe(II). The ratio of maximum oxidation of solid Fe(II) to aqueous Fe(II) oxidized is comparable for both experiments with a ratio of 2.20 for the experiment of the main manuscript and 2.89 in the here presented dataset.

Figure S17: Geochemical results from further chemostat run with culture KS. For Fe: circles show Fe(II) species while squares show Fe(III) species for aqueous (aq – grey) and solid phase (s – orange) iron (mmol L^{-1}). Nitrate is shown as black circles. Since culture KS was not pre-grown for this experiment, we recorded a much greater lag phase, as seen by the fluctuating/stagnating Fe(II) values.

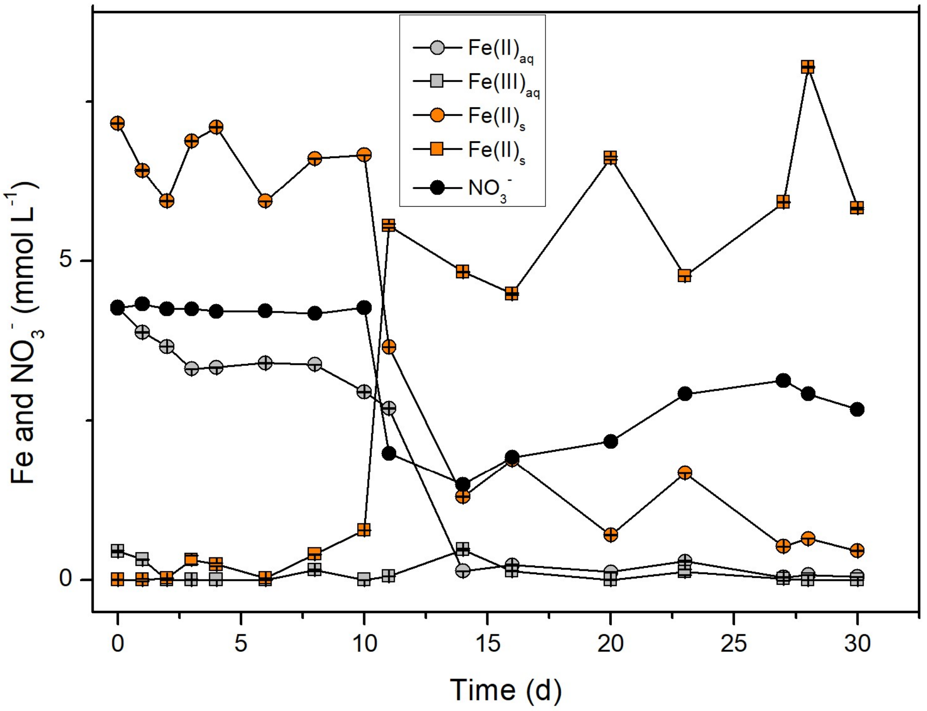

Table S1. Recorded values for Mössbauer spectroscopy measured at 77 K for samples collected from the chemostat during continuous cultivation of autotrophically grown culture KS with 10 mM Fe(II) and 4 mM of nitrate. δ – isomer shift, ΔE_Q – quadrupole splitting, $\sigma(\Delta E_Q)$ – standard deviation of ΔE_Q, R.A. – relative spectral area, Reduced (Red.) X^2 – goodness of fit.

Sampling timepoint (days)	Oxidation state	δ (mm/s)	ΔE_Q (mm/s)	$\sigma(\Delta E_Q)$ (mm/s)	R.A. (%)	error (%)	Red. X^2
0	Fe(II)	1.34	2.56	0.46	71.3	6.2	0.57
	Fe(II)	1.32	3.26	0.03	22.7	6.3	
	Fe(III)	0.38	0.79	0.17	6.0	1.9	
3	Fe(II)	1.25	3.00	0.18	9.2	1.3	0.75
	Fe(III)	0.49	0.79	0.30	90.8	1.3	
7	Fe(II)	1.23	3.09	0.25	7.3	2.1	0.69
	Fe(III)	0.49	0.80	0.31	92.7	2.1	
14	Fe(II)	1.26	2.95	0.28	18.3	4.0	0.63
	Fe(III)	0.50	0.75	0.29	81.7	4.0	
24	Fe(II)	1.15	2.84	0.13	5.1	2.9	0.60
	Fe(III)	0.49	0.82	0.33	94.9	2.9	

T**able S2:** Results of unpaired t-test for differences treatments.

Phase	Comparison	t	df	p-value
NA	Aqueous vs solid	-3.4161	13.589	0.00434
Aqueous	Big vs small	1.2745	8.6747	0.2356
Aqueous	Static vs shaking	-1.0946	8.8422	0.3026
Solid	Big vs small	-2.5091	9.9184	0.03114
Solid	Static vs shaking	1.7128	9.8305	0.1181

Table S3: Calculated rates of the conducted run before the study of the main manuscript in the chemostat (see Figure S17).

Setup	Fe(II) oxidation (mM d^{-1})	
	Aqueous Fe(II)	*Solid phase Fe(II)*
Chemostat (previous run)	1.12	3.27

References

Daugherty, E.E., Gilbert, B., Nico, P.S., and Borch, T. (2017) Complexation and Redox Buffering of Iron(II) by Dissolved Organic Matter. *Environ Sci Technol* **51**: 11096-11104.

Klueglein, N., Zeitvogel, F., Stierhof, Y.D., Floetenmeyer, M., Konhauser, K.O., Kappler, A., and Obst, M. (2014) Potential role of nitrite for abiotic Fe(II) oxidation and cell encrustation during nitrate reduction by denitrifying bacteria. *Appl Environ Microbiol* **80**: 1051-1061.

Schaedler, F., Kappler, A., and Schmidt, C. (2017) A Revised Iron Extraction Protocol for Environmental Samples Rich in Nitrite and Carbonate. *Geomicrobiol J* **35**: 23-30.

Segre, C., Leyarovska, N., Chapman, L., Lavender, W., Plag, P., King, A. et al. (2000) The MRCAT insertion device beamline at the Advanced Photon Source. In *AIP Conference Proceedings*: American Institute of Physics, pp. 419-422.

Shimizu, M., Zhou, J., Schröder, C., Obst, M., Kappler, A., and Borch, T. (2013) Dissimilatory Reduction and Transformation of Ferrihydrite-Humic Acid Coprecipitates. *Environ Sci Technol* **47**: 13375-13384.

Stookey, L.L. (1970) Ferrozine - a new spectrophotometric reagent for iron. *Anal Chem* **42**: 779-781.
